# Perinone—New Life of an Old Molecule

**DOI:** 10.3390/ma14226880

**Published:** 2021-11-15

**Authors:** Mieczysław Łapkowski

**Affiliations:** 1 Faculty of Chemistry, Silesian University of Technology, Strzody 9, 44-100 Gliwice, Poland; mieczyslaw.lapkowski@polsl.pl; 2 Centre of Polymer and Carbon Materials, Polish Academy of Sciences, 34 M. Curie-Sklodowska Street, 41-819 Zabrze, Poland

**Keywords:** perinone, synthesis, photochemical properties, electrochemistry, organic electronic

## Abstract

A review of publications on the synthesis and properties of a family of compounds called perinones was carried out. The basic molecule has been known for several decades mainly as a photostable pigment, and in recent years it has become increasingly used in organic electronics. This paper describes the methods of synthesis of low molecular weight compounds and polymers based on that molecule; the basic spectroscopic, photochemical, electrochemical and electronic properties important for the construction of organic electronics and optoelectronics devices are also discussed.

## 1. Introduction

**Scheme 1:**
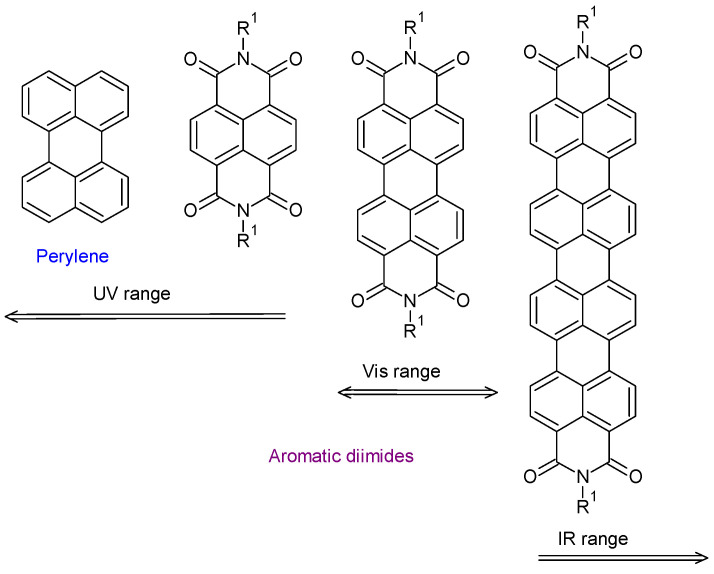
Color change of diimide dyes as a function of extension of aromatic core.

**Figure 1:**
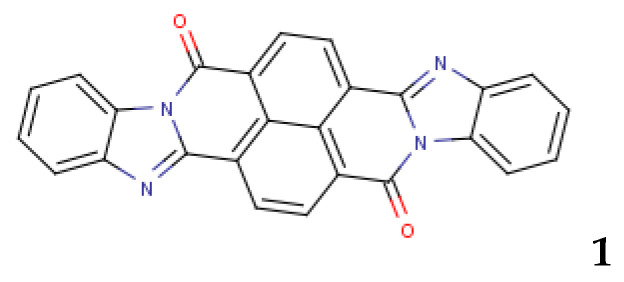
Perinone chemical structure. (Bisbenzimidazo[2,1-b:2′,1′-i]benzo[lmn][3,8] phenanthroline-8,17-dione).

Organic electronics have been developing intensively for at least 30 years due to their promising advantages; low material and production costs are the most important among them. It is based on conjugated compounds and polymers occurring in non-doped or semiconductor form which can often be processed in solution under ambient conditions. The organic semiconductors are generally characterized by light weight, ease of molecular design and flexibility, compared with classical inorganic materials. These properties allow a wide development of print-production technologies for the scale-up production of organic electronic and photonic devices under the concept of printed electronics technologies [1] and makes them competitive for the production at low cost and for large-area devices in a variety of electronic applications such as supercapacitors, solar cells, ambipolar field-effect transistors, and light-emitting and electrochromic devices [2,3,4]. For that purpose, thiophene, benzene, triarylamine, and carbazole derivatives dominate. These materials are already incorporated into curved displays, such as television and smartphone screens in the form of organic light-emitting diodes (OLEDs). In pursuit of higher stability, new low-molecular weight compounds, polymers, and copolymers are intensively investigated. One way to improve stability is to take inspiration from things found in nature, which as a result of evolution has selected many chemical compounds that are stable in natural conditions and resistant to solar radiation. This is especially important when using organic compounds to build photovoltaic diodes. These compounds primarily include porphyrin derivatives [5,6,7], which are components of the structure of chlorophyll [8], and indigo [9,10], which is an extremely persistent naturally occurring dye, known for millennia [11]. These studies led to the discovery of synthetic counterparts of these compounds with comparable properties, such as phthalocyanines [12,13], isoindigo [14,15], and aromatic diimides [16,17], of which the most important are perylene dyes [18,19]. The last of these compounds have made a substantial career in the synthesis of dyes used for fabric dyeing, and in the production of automotive paints. In both examples, the high resistance to sunlight is of great importance here. The expansion of planar π-conjugation of arylene cores improves many physicochemical properties significant in organic electronic application, such as charge carrier mobility, stability, or optical properties [20,21,22]. The new planar imidazole ring addition to the aromatic perylene core provides longer wavelength absorption, which however, reduces solubility of the product, as shown in Scheme 1 [23,24]. Compounds obtained by condensation of o-phenylenediamine with naphthalene tetracarboxylic give a perinone molecule, as shown in Figure 1. Modification of perylene pigments (Scheme 1) offers a relatively simple way to synthesize compounds with absorbance covering the whole range of the visible spectrum.

Perinone is a molecule obtained in the laboratory of Hoechst in Frankfurt-on-the Main, Germany, over ninety-five years ago, [25,26,27,28] and it was used as a pigment in the dye industry [29,30,31]. Studies summarizing these applications have been published systematically over the past 30 years [23,32,33,34]. Its name according to IUPAC is bisbenzimidazo[2,1-b:2′,1′-i] benzo[lmn][3,8]phenanthroline-8,17-dione; its structure is shown in Figure 1.

**Figure 2:**
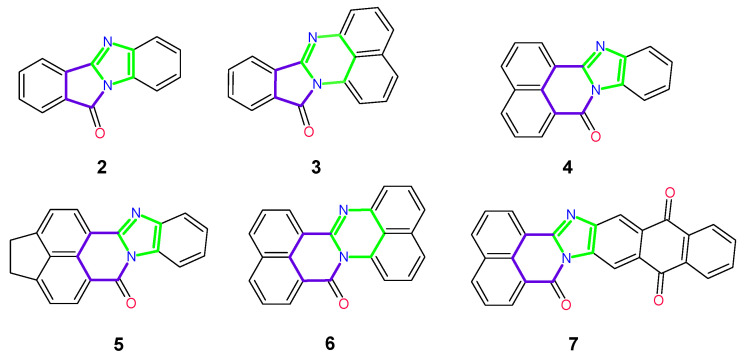
Sample structures of small perinones.

It should be noted, however, that similar compounds were obtained a little earlier, but these were obtained by using aromatic dicarboxylic acids in the condensation process with aromatic diamines [35,36]. Exemplary structures are shown in Figure 2.

**Figure 3:**
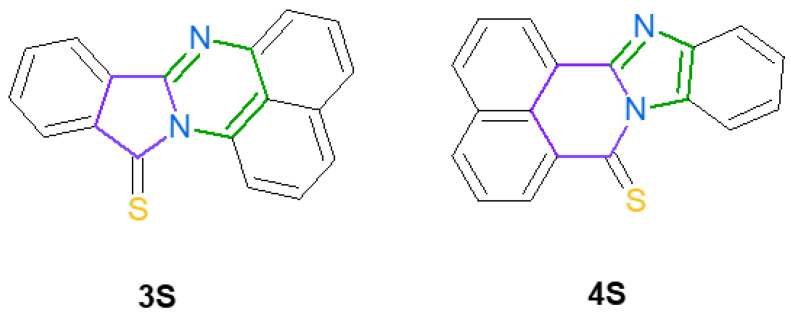
Sample structures of small thioxo-derivatives of perinone.

There are also known thioxo-derivatives shown in Figure 3 [37].

The functional group of perinone derivatives is composed of two conjugated heterocyclic rings containing nitrogen atoms, marked in purple and green. The sizes of these rings are different since they can be five- or six-membered, hence we have four combinations: in molecule 2 (pyrrolo-2-one and imidazole), molecule **3** (pyrrolo-2-one and pyrimidine), six- and five-membered rings in molecules **4**, **5,** and **7** (pyridin-2-one and imidazole), and two six-membered rings in molecule **6** (pyridin-2-one and pyrimidine). All structures are flat, hence the perinone derivatives tend to form π-stacks.

## 2. Synthesis

**Figure 4:**
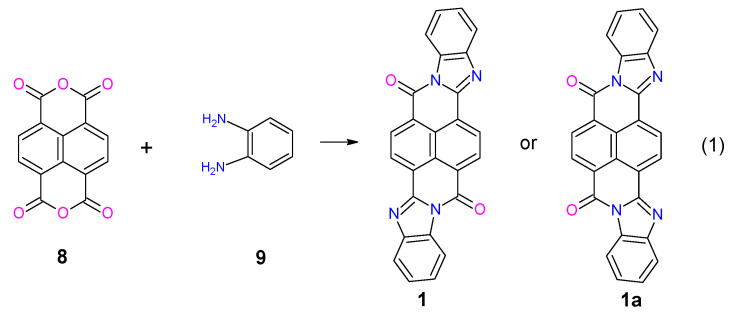
Typical reaction scheme for synthesis of perinone trans- (**1**) and cis- (**1a**) isomers.

The perinone system is usually synthesized by condensation of monoanhydride or dianhydride naphthalene tetracarboxylic acid (**8**) with o-diaminobenzene (**9**) in glacial acetic acid at 397 K [21,38]. The reaction scheme is shown in Figure 4.

The reaction (1) leads to a mixture of both trans and cisperinone isomers (**1** and **1a**, respectively) at a ratio of approximately 1:1 with a slight preference for the transisomer. These isomers are insoluble in the reaction mixture, and thus they precipitate in a form of mixed crystal. Their separation occurs via differences in the solubility of their respective salts. The trans isomer can be precipitated as a sparingly soluble colorless potassium ring opening compound, by heating the mixture in ethanol/potassium hydroxide solution. Good results are also obtained through fractionation with 90% sulfuric acid. Last one of these techniques is followed by the traditional method of converting the products into commercially useful pigments. The options include milling, acid treatment, and solvent treatment at elevated temperature. A detailed description of the isomer separation along with the description of possible intermediates and their crystalline structures can be found in the new work of Tapmeyer et al. [34]. The perinone pigments are commercially available as, for example: Indanthrene Scarlet GG (mixture of both isomers), Indanthrene Brilliant Orange GR (trans isomer) and Indanthrene Bordeaux RR (cis isomer) or Vat Red 14 (mixture of both isomers), Pigment Orange 43 (trans isomer), and Pigment Red 194 (cis isomer). Taublaender et al. [39] prepared perinone hydrothermally, using high-temperature water (typically 180–250 °C) at the starting compounds in a stoichiometric ratio. This green method of synthesis is highly robust and remains unaffected by various reaction parameters, it also does not require condensation promotors or catalysts. Using this method, it is also possible to crystallize previously obtained amorphized perinone or recrystallize commercially obtained perinone.

Synthesis of thioxoderivatives of perinone **3S** and **4S** can be realized by condensing trithioanhydrides and appropriate diamines at relatively high yields. Direct sulfuration of the corresponding molecules (**3**, **4**) did not lead to the intended products in a synthetically useful yield [40,41].

**Figure 5:**
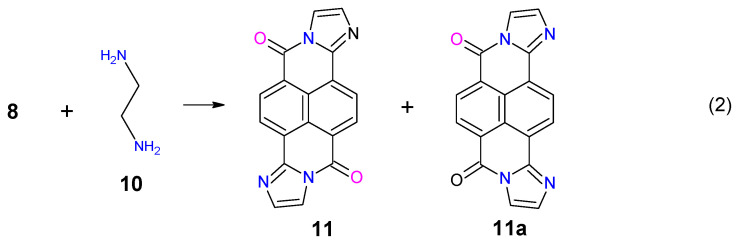
Scheme of synthesis of benzo[lmn]bisimidazo[2,1-b:2′,1′-i]phenanthroline-6,12-dione (**11**) and benzo[lmn]bisimidazo[2,1-b:2′,1′-j]phenanthroline-3,6-dione (**11a**) [42].

The condensation of dianhydride naphthalene-1,8:4,5-tetracarboxylic acid (**8**) with ethylenediamine (**10**), shown in Figure 5, led to isomeric bisamidines **11** and **11a**, characterized by formation of two imidazole moieties. The mechanism of aromatization of imidazole rings was not clearly explained.

Another way to obtain **11** and **11a** was proposed in transamination reaction in which naphthalene bisimide reacted with ethylenediamine [42]. These two molecules can be treated as shorter analogues of trans and cisperinones (**1** and **1a**). Isomers **11** and **11a** are sufficiently soluble to be used in homogeneous solution and can be separated by column chromatography. The transisomer (**11**) is yellow with absorption maximum located at about 450 nm when the cisisomer (**11a**) is red with the maximum at 510 nm. The different absorption spectra for these isomers correspond to the different colors of the benzo analogues of **1** and **1a**, 480 nm and 535 nm respectively. Compound **11** fluoresces with a quantum yield of 40% and a Stokes shift of 100 nm, whereas no fluorescence was detected for the more bathochromically absorbing **11a** [42].

**Figure 6:**
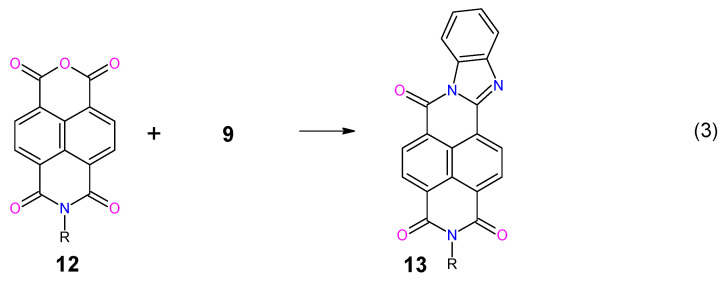
Synthesis of unsymmetrical perinone **13**.

Due to the insolubility of **1** and **1a** in typical solvents, it is also possible to obtain an unsymmetrically annulated imidazo cycle, in which bulky alkyl substituents in the imide part of molecule provide solubility. Such molecules can be obtained by the reaction of aromatic monoimides with o-phenylene diamine or its derivatives, as shown in Figure 6 [43,44].

**Figure 7:**
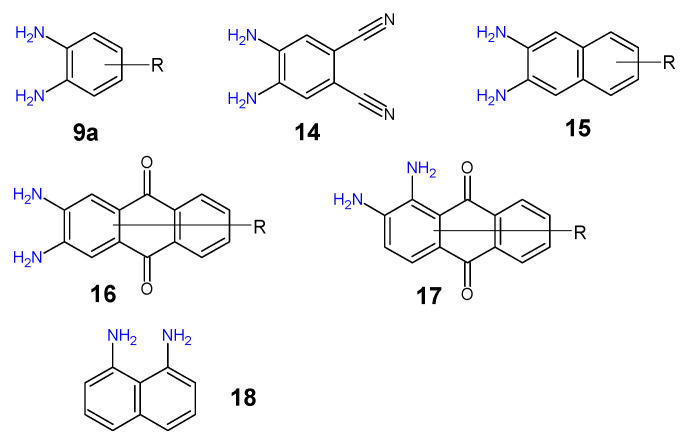
Selected aromatic diamines used for perinone synthesis [44,45].

Asymmetrical perinone derivatives of **13** were obtained by the reaction shown in Figure 6, mainly by changing aromatic diamines. The most commonly used diamines are shown in Figure 7 [44,45].

Kobrakovet et al. [46] showed another way to synthesize unsymmetrical perinone derivatives by direct reaction of ortho or peri-tetracarboxylic acids, or the corresponding dianhydrides, with aromatic ortho- or peri-diamines. In the first step, only one imidazole ring is formed whereas the second anhydride group is preserved in the structure of the dye for further chemical modification of the molecule to obtain an assortment of dyes with a new set of properties. Using this method they synthesized 28 thermostable different dyes with a wide range of colors, from light yellow through purple to dark blue.

**Figure 8:**
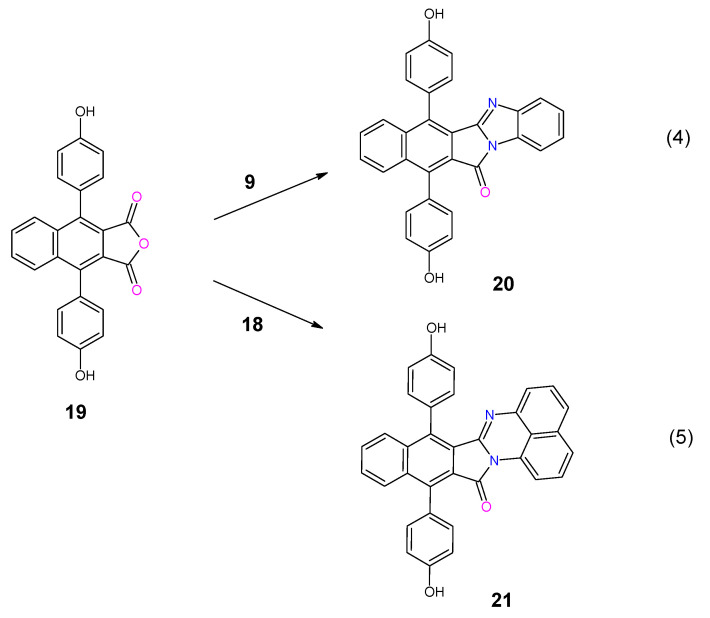
Synthesis scheme of bisphenol derivatives of perinone [47].

Meng et al. [47] synthetized two bisphenols containing a benzoylene-benzimidazole moiety, **20**, and a phthaloperinone moiety, **21**, from the anhydride **19** by reaction with aromatic diamines as shown in Figure 8.

There is no melting point for molecule **20** whereas molecule **21** has a melting point of 418.7 °C with decomposition. Perinones **20** and **21** are yellow and orange-red in color, respectively, and they are both highly fluorescent. The bisphenol hydroxyl groups allow the use of both molecules to obtain classic polyethers. The two phenolic moieties are almost perpendicular to the planes of the very bulky heterocyclic moieties. The authors say that the random arrangement **20** and **21** unsymmetrical monomers in poly(aryl ether)s might therefore be expected to enhance the solubility of the polymers [47].

**Figure 9:**
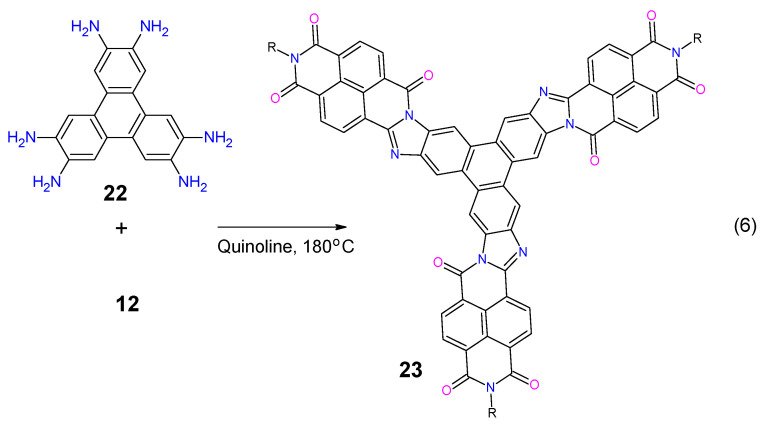
Synthesis scheme of disc-shaped perinone derivatives **23**.

Zhang et al. [48] synthesized a C_3_-symmetric disk-shaped molecule containing a triphenylene core fused with three naphthaleneimide imidazole “arms”. The molecule was obtained as an inseparable statistic mixture of cis (symmetric) and trans (asymmetric) isomers, with a ratio of 1:3, (both displaying a good solubility in solvents such as CH_2_Cl_2_ and CHCl_3_) by the condensation reaction between hexaaminotriphenylene (**22**) and the corresponding naphthalene mono-anhydride (**12**) bearing an n-alkyl chain [49]. The synthesis scheme is shown in Figure 9.

Extended conjugation of this type of molecule led to enhanced optical properties along with well-aligned frontier orbital energies. Moreover, charge carrier mobilities show drastically different directional anisotropy and atomic force microscopic analyses revealed a strong correlation between the film morphology and the charge transport behavior. The molecules are arranged into ordered hexagonal packing with edge-on orientation in thin films [48,50]. The charge transport in discotic systems is highly influenced by the local arrangement of stacked π-conjugated cores and the length, type (linear/branched), and chemical structure of the side chains [51,52]. In the case of molecule **23**, experimental results indicate that the use of linear side chains instead of branched ones can effectively enhance the order within the thin films and, correspondingly, the electron-transport properties. In solid film, a twisted columnar stacking with a rotation angle of around 13° was obtained. The deviation from cofacial stacking is highly detrimental for charge mobilities due to the smaller overlap of the electronic wave function of the two molecules in the dimer and more so for electron-transport mobility such that the dominant carriers become holes instead of electrons [49].

**Figure 10:**
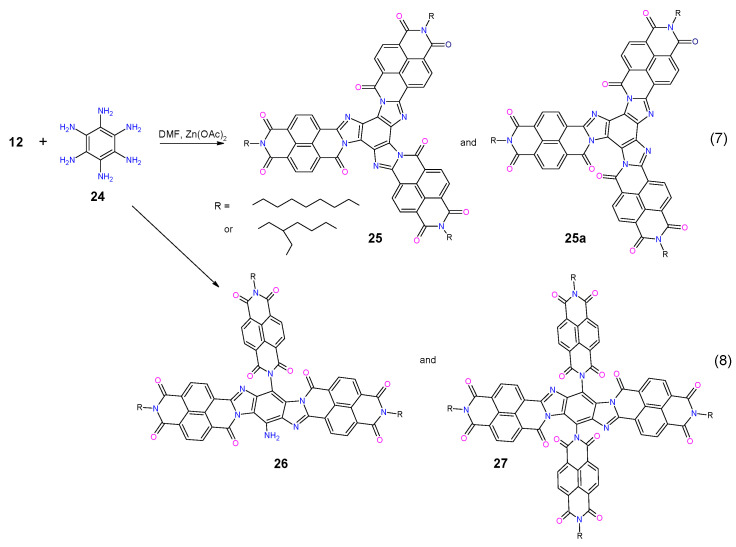
The scheme of condensation reaction for monoanhydride naphthalene-1,8:4,5-tetracarboxylic acid monoimide (**12**) and hexaaminobenzene (**24**).

Another way to obtain C_3_-symmetric disk-shaped molecules was presented by Hanifi et al. [53]. They attempt to synthesize this type of molecule by using a hexaaminobenzene (**24**) in condensation reaction. The synthesis reaction is shown in Figure 10.

The reaction is not a simple condensation as reaction (7) but it leads to several soluble products. Orange, green, and deep red fractions were isolated and analyzed using NMR spectroscopy. The red product **25** has the same mass as **25a** and can be assigned to be a cyclic trimer isomer. The orange and the green adducts was assignable to the acyclic trimer **26** and the C_2_-symmetric tetramer **27**, respectively. The extended conjugation in **25** promotes strong π–π intermolecular interactions and supramolecular aggregation was observed not only in solid state but also in solution. As a result, a phase-separated precipitate is formed from its CHCl_3_ solution within one day upon standing at room temperature. The crystals were formed in highly entangled nanofibers due to anisotropic stacking of **25** by slipped π–π cofacial stacking [53].

**Figure 11:**
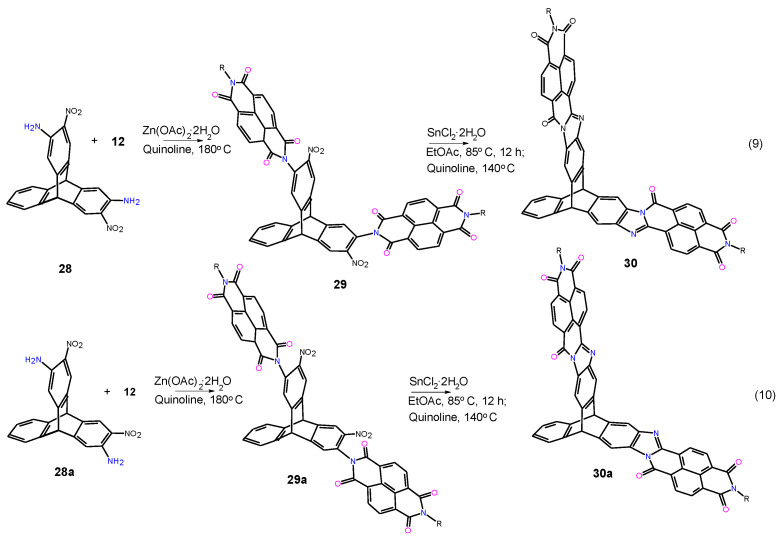
Synthetic road to C_2_ and Cs—symmetrical perinone–triptycene molecules.

To avoid intrinsically large stacking energies of flat perinone derivatives, Menke et al. [54,55] decided to attach aroyleneimidazole units to a triptycene core. Finally, they received a number of bis- and tris-substituted stereoisomers as a result of clever control of the position of nitro and amino groups on the triptycene molecule (**28** and **28a**). An exemplary synthesis of disubstituted perylene–triptycen derivatives and its condensation to perinone–triptycene derivatives is shown in Figure 11 [55].

The color of all stereoisomers **30** and **30a** is the same with the maximum absorption peak located around 484 nm and luminescence peak at 616 nm. The electronic influence of alkyl substituents at the imide-N atom is negligible and the spatial arrangement of the two aroyleneimidazole units does not differ in homoconjugation between these units. Application of these molecules to OPV devices shows that molecular symmetry plays a role in the overall performance. A lower symmetry number of the molecular acceptor results in a higher performance [55].

**Figure 12:**
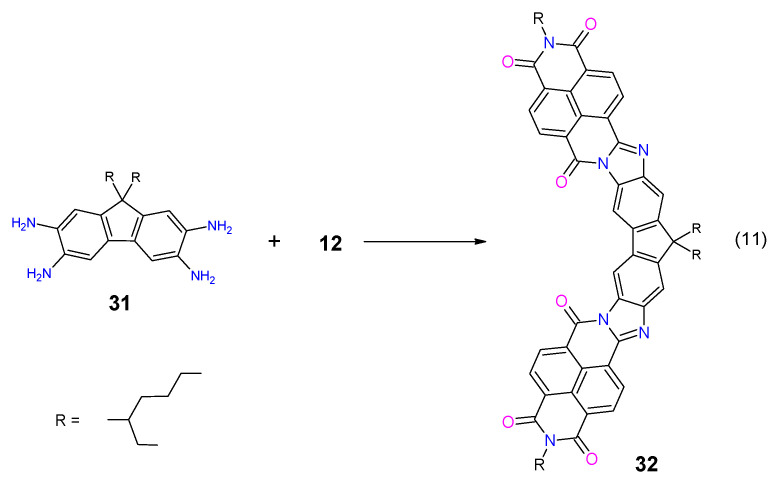
Synthesis reaction scheme for symmetrical bisperinone derivative **32 [56]**.

Another way to increase the solubility of perinone derivatives was to use aromatic diamines or tetraamines substituted with long or branched alkyl chains. Li et al. [56] applied 2,3,6,7-tetraamino-9,9-bis(2-ethyl-hexyl)fluoren (**31**) in condensation reaction with **12** for synthesis of symmetrical bisperinone derivatives (**32**), as shown in Figure 12.

Even under relatively mild conditions (reflux in acetic acid) this reaction proceeded readily. The products were obtained as a mixture of two isomeric molecules (unsymmetrical and symmetrical), which were successfully isolated via column chromatography. These molecules exhibit very strong absorption across a broad wavelength range and show distinct red shift in absorption maxima and significant increase in absorption intensity, compared with the bisiimide analog.

**Figure 13:**
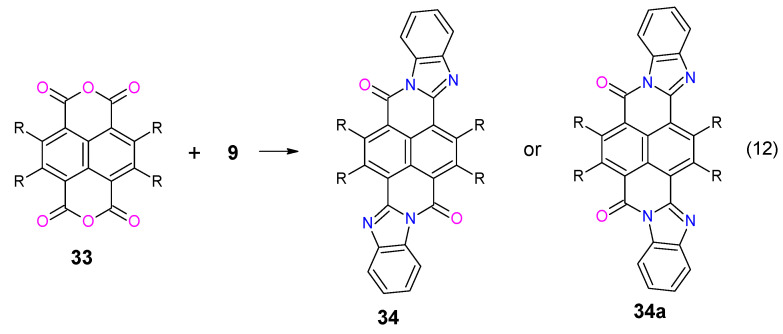
Reaction scheme of o-phenylenediamine (**9**) with 2,3,6,7-tetraoctyl-1,4,5,8-naphthalene tetracarboxylic bisanhydride (**33**). R = n-octyl.

A different strategy to obtain perinone solubility was used by Xiong and Xiao [57]. As a result of a multi-stage process, they synthesized an alkyl derivative of bisanhydride (**33**) and then condensed it with o-phenylene diamine (**9**), as shown in Figure 13.

In contrast to perinone isomers (**1**, **1a**), both **34** and **34a** molecules are well soluble in typical solvents such as chloroform and dichloromethane. The absorption spectrum was vibrionic in character with the maximum located at about 460 nm. Both showed broad emission band centered at 549 nm and exhibited a high fluorescence quantum yield of 0.47.

**Figure 14:**
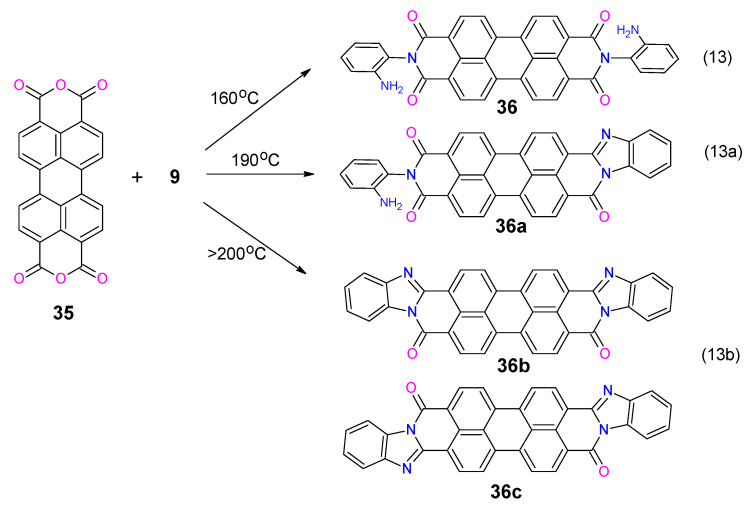
Condensation of perylenetracarboxylic acid dianhydride (**35**) with **9** at different temperatures.

The extension of the aromatic core in tetracarboxylic dianhydrides is accompanied by the continuous bathochromic shift of the absorption and fluorescence emission bands as a consequence of the extension of the conjugated π system along the long axis. The next member of this family is 3,4,9,10-perylene-tetracarboxylic anhydride (**35**). Condensation of **35** with o-phenylenediamine (**9**) was temperature dependent and at elevated temperature gave non centrosymmetric cis and symmetric trans perinone derivatives (**36b** and **36c,** respectively) [58]. The scheme of the reaction is shown in Figure 14.

At lower condensation temperature (160 °C), the main product (67.1%) was N, N′-di (o-amino) phenyl-perylenetetra-carboxyl-diimide with 2 amine groups free (**36**) (reaction13), which is soluble in alkaline hydrosulfite and insoluble in 90% sulfuric acid. The color of solution was dull-red violet of low value as a vat dye. At moderate condensation temperature (190 °C), a 3,4-benzimidazole-9,10-(o-amino)phenyl-perylenetetracarboxyl-diimide (**37**) with one free amine group was obtained with a yield (60.6%). This nonsymmetric derivative of perinone **36a** was easily soluble in alkaline hydrosulfite and also in 90% sulfuric acid giving a solution having a deep violet shade with a strong dyeing power. At temperatures higher than 200 °C, the main product was 3,4,9,10-perylenetetracarboxyl-bis-benzimidazole (**36c**), a symmetric perinone, which was a violet blue substance insoluble in alkaline hydrosulfite and in common solvents. The diamino compound (**36**) could be converted mainly into (**36a**) by boiling with nitrobenzene for 1~1.5 h and it almost entirely changed into the perinones (**36b**, **336c**) when boiled with nitrobenzene for 5 h [58]. The insolubility of the molecule has proved to be an obstacle to its wider use, and in the dye industry, but long-wave absorption properties can be used to produce IR absorbing pigment [59].

**Figure 15:**
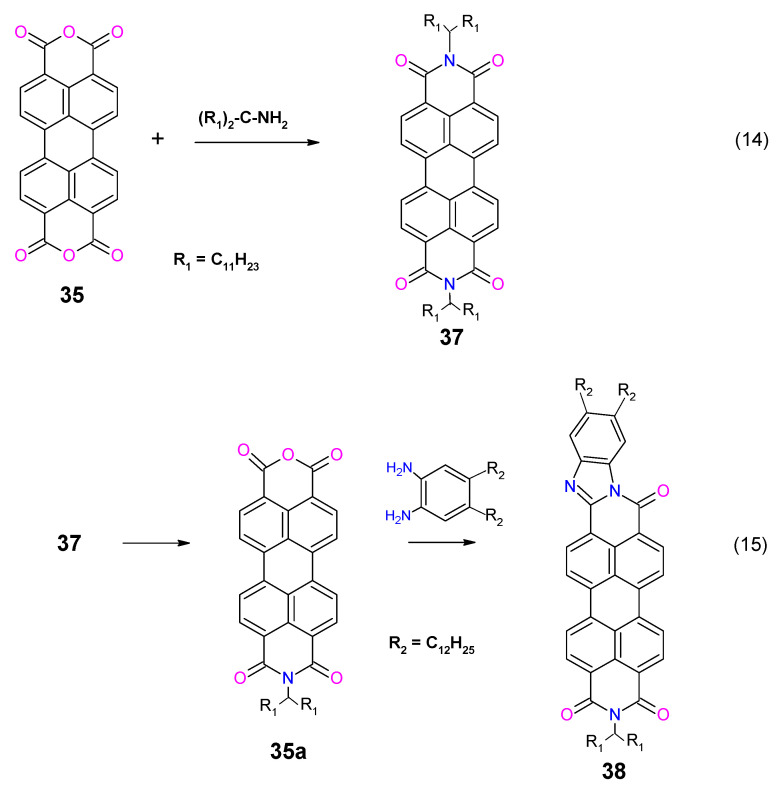
Synthesis of asymmetric perylene imide benzimidazole [60].

Wicklein et al. [60] described the synthesis of an asymmetric perylene imide benzimidazole using **35** and 1,2-diamino-5,6-dialkybenzene, as shown in Figure 15.

In the first step, N-substituted perylene bisimide 37 was hydrolyzed with potassium hydroxide in tert-butyl alcohol allowing access to imide-anhydride **35a**. The benzimidazole moiety was subsequently introduced by condensation of 1,2-diamino-phenyl with obtained monoimid–anhydride in molten imidazole solution. They attached an alkyl swallow-tail substituent at the imide nitrogen atom and two long alkyl chains at the benzimidazole unit in order to guarantee sufficient solubility of molecule **38**. This nonsymmetric perinone derivative tends to self-assemble into columnar structures and appears to display an extended absorption range compared with conventional perylene bisimides. The absorption maximum was located at 556 nm with a very high molar extinction coefficient; ε = 4.8 × 10^4^ L mol^−1^cm^−1^. This mesogen perinone derivative **38** self-organizes into a hexagonal columnar liquid crystalline phase at high temperatures and transforms into a columnar plastic phase at lower temperatures.

The use of aromatic diamine derivatives such as 4-chloro-1,2-phenylenediamine, 4-methoxy-1,2-phenylenediamine, or 1,2-naphthylenediamine in the reactions (13–15) shown in Figure 13 leads to the corresponding derivatives of **36**, **37, 38,** and **38a** using the same processing parameters. The obtained chloro and methoxy derivatives of perylene diimides or perinones have similar properties with their color slightly changed. For example, the dichloro-noncentrosymmetric cis perinone derivative was a black violet powder, soluble in concentrated sulfuric acid, whereas the solution shows blue (thin layer) and violet blue (thick layer) dichroism with weak red fluorescence. The similar methoxy-derivative was a dark violet powder. The concentrated sulfuric acid solution was blue (without dichroism) displaying weak red fluorescence [61].

**Figure 16:**
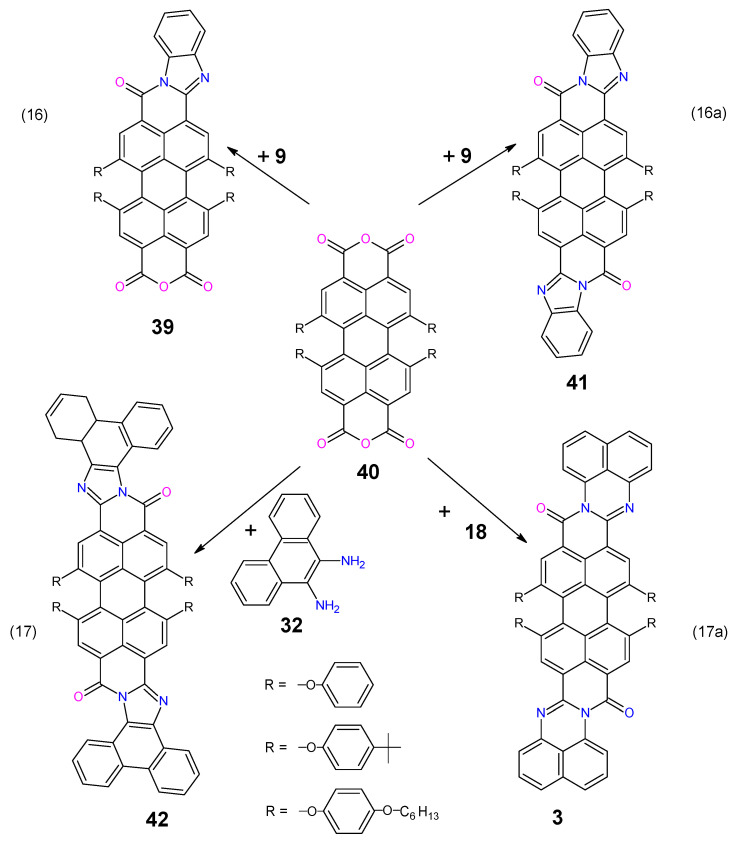
Synthesis schemes of monoarylamidineperylenes **39** and bisarylamidineperylenes **41–44**, substituted in the bay region of the perylene core. Quinoline solution at 220 °C [59].

The solubility problem of perylene perinones was solved as in the case of naphthalene bisimides by attaching large substituents in the bay region of the perylene core. Using this approach, Müllen et al. [59] prepared a number of perinones shown in Figure 16.

All of these molecules had long-wavelength absorption and emission spectra with fairly high extinction coefficient, good solubility in organic solvents, as well as high thermal stability. With short reaction time (45 min), monoimidazole perylene (**39**) was obtained as a side product at low yield (16%), from the condensation of dianhydride **40** with an equimolar amount of o-phenylenediamine (**9**), whereas the cis and trans perylenes mixtures, **41**, were obtained at 62% yield. The yield of **41** was increased to 87% when a 3.3-fold excess of **9** and a reaction time of 4 h were applied. Similar reaction conditions were used for the syntheses of **43**, and **44**, resulting in high yields (72–88% of cis and trans perylene derivatives). Despite their extended rigid molecular structures, solubilities of all molecules in common organic solvents were good. The extension of the π-system induces a dramatic bathochromic shift compared with their bisimide analogs. The bisnaphthalene condensation product **44** and the bisphenanthrene condensation product **43** exhibit longer wavelength absorption maxima of 652 and 659 nm, respectively [59]. The optical band gap also changes significantly compared with diimides, decreasing in the case of the **43** molecule by 0.2 eV, and in the case of the **44** molecule by 0.33 eV. The significant reduction in band gap in the case of the **44** molecule can be explained by the presence of amidine six-membered rings, whereas in the case of **43**, amidine subunits are incorporated in five-membered rings. Perinones **38**, **41,** and **43** have good fluorescent properties, while for the **44** molecule fluorescence is weak, probably due to possible overlapping between their very broad absorption and emission bands which results in the reabsorption of the emitted light by the chromophore itself [59]. It should be noted that substitution with tetra-phenoxy groups at the bay region induced a decrease in the electron-accepting properties due to their predominant electron-donating effects [62]. Use of perylene monoimide instead of **39** in the synthesis process shown in Figure 16 led to unsymmetrical perinones which can easily be obtained [33,43,44,63,64].

**Figure 17:**
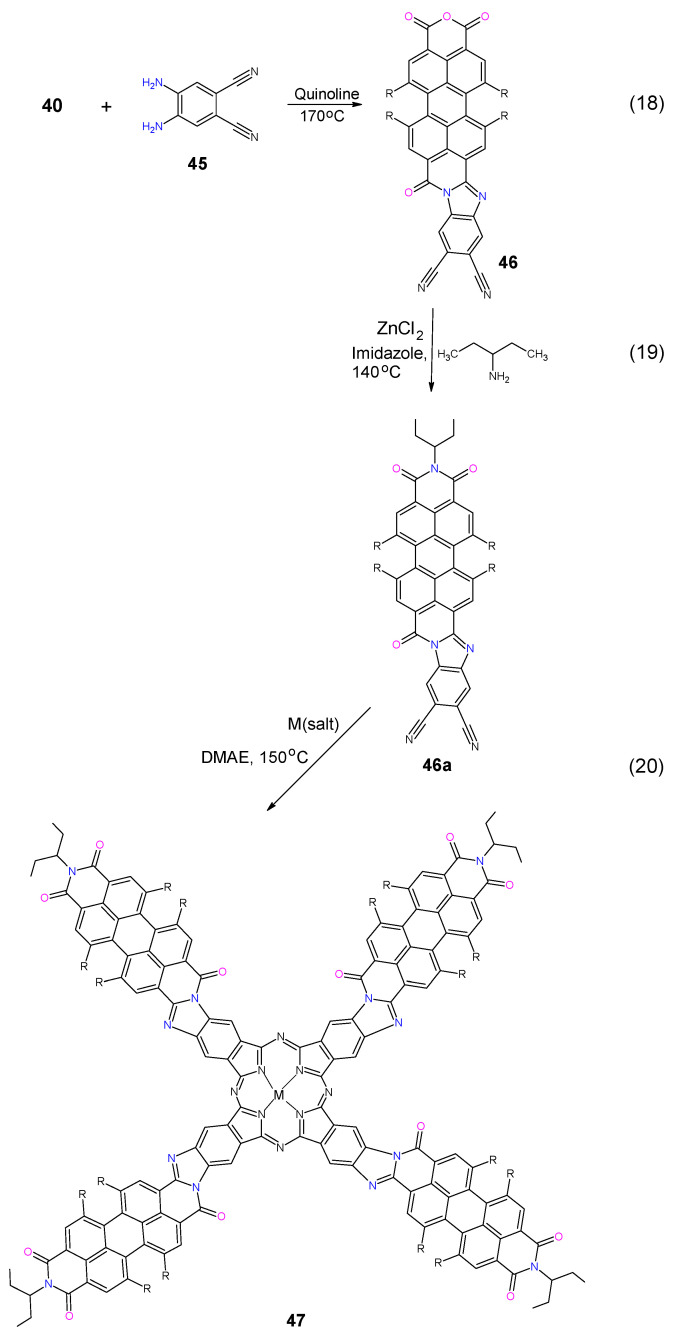
Synthesis scheme of perylene–phthalocyanine hybrids (**47**) [65].

Using **40** with the tert-butylphenoxy group as R-substituents on perylene core, Schönamsgruber et al. [65] synthesized a soluble perylene–phthalocyanine hybrid **47**. The synthesis was a multistep process presented in Figure 17.

In the first step, monoanhydride–monobenzimidazolide perylene (**46**) was obtained at 20% yield. In the next step, the remaining anhydride position of **46** was condensed with 3-aminopentane in molten imidazole to afford the imide **46a** in 90% yield. The obtained dinitrile **46a** was starting material for the synthesis of phthalocyanines (Pc) in the last step according to the procedure developed by Kimura et al. [66]. Despite the flat multiaromatic molecular structure, **47** was well soluble in typical solvents, which allowed for a thorough analysis of the obtained structures. Concerning the relative orientation of the benzimidazole-like bridges between the perylene moiety and the Pc core, four different isomers are conceptually possible (neglecting the conformers due to different orientations of the bay substituents and perylene plane twisting), but isomers with C_4h_ symmetry were formed almost exclusively. Steric hindrance caused by the bulky bay-substituents of the perylene subunits was minimized when the macrocycle formation of the carbonyl O atoms are preferentially forced to point in the same direction, giving a molecule with C_4h_ symmetry. The extensive fusion of the corresponding aromatic building blocks to a very large extended π-system led to a very narrow HOMO–LUMO gap and as a consequence to transparency in the visible, but light absorption in the NIR region. Protonation shifts the absorption maximum back to the visible region due to the loss of conjugation. The azomethine N-atoms in Pc central ring and the corresponding tetraprotonated **35** PcH_4_^+^ systems can only be deprotonated with very strong non-nucleophilic bases such as phosphazene bases. The most intensive absorption band in UV–Vis spectrum of **35** PcH_4_^+^ appears at 450 nm (ε = 3800 L mol^−1^cm^−1^), 560 nm (ε = 8800 L mol^−1^cm^−1^), and 602 nm (ε = 13,800 L mol^−1^cm^−1^). The emission band is located at 655 nm (excitation at 600 nm). The shape of the emission spectrum reflects a mirror-image behavior with respect to the absorption spectrum. In the deprotonated **47**Pc molecule, three bands are also present at 812, 1002, and 1109 nm with increased molar absorption (22,000 L mol^−1^cm^−1^ at 812 nm). The protonation and deprotonation processes were fully reversible, so it offers a molecular NIR switch triggered by controlled acid and base addition [65].

**Figure 18:**
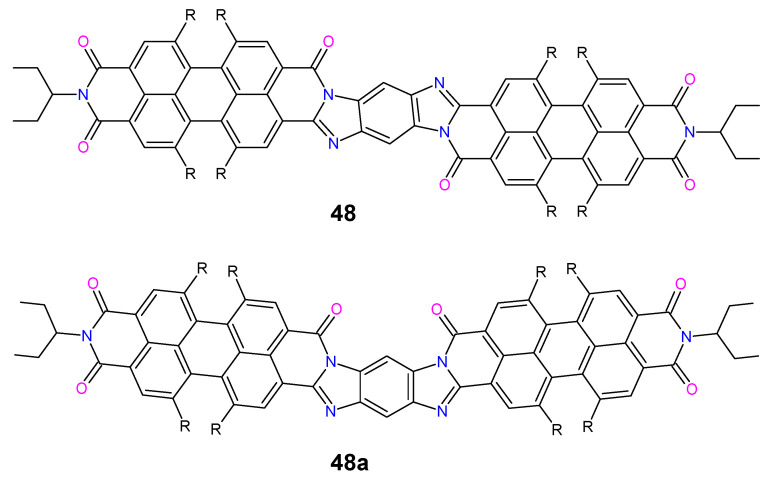
The linear imide fused stereoisomers linked by benz-bisimidazole substructures in the center (**48, 48a**) [65].

By replacing **45** by 1,2,4,5-tetraaminobenzene tetrahydrochloride in the reaction (18), the linear compounds **48** and **48a** shown in Figure 18 were obtained [67].

In these architectures, the perylene units are linked by a benz-bisimidazole substructure in the center. These perinone derivatives exhibit rather extended linear structures with 35 conjugated π-electron pairs. Two isomers appear during the synthesis with an “anti” (**48**) or “syn” (**48a**) configuration of the bridging imide groups relative to each other.

**Figure 19:**
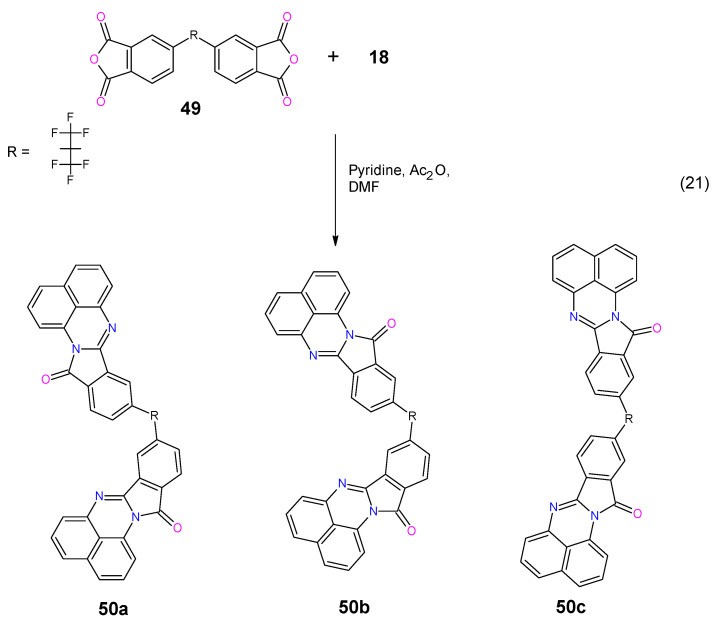
Synthetic route giving the mixture of three isomers of perinone derivatives.

The formation of various structural isomers during condensation is often ignored but their physicochemical properties could influence noncovalent interactions such as hydrogen bonding [47], dipole–dipole interactions [68], π–π stacking, as well as chiral recognition [68]. Therefore, when designing new perinones, it is important to consider the possibility of obtaining various isomers, which despite their highly similar structures, may have different properties, important for organic electronic applications. 4,4′-R-diphthalic anhydride is the typical monomer used for synthesis of thermostable polyimides and was used for synthesis of perinone derivatives. Due to its structure, during condensation of o-diaminobenzene or 1,8-diaminonaphthalene, three isomers could be obtained in the reaction shown in Figure 19.

The obtained deep-red powder was a mixture of three isomers (**50a–c**) soluble in common organic solvents. The chromatographic separation of the mixture allowed separation of the isomers and permitted the determination of their content in the original mixture, which was approximately 40% for isomer **50a** (9,9′ isomer), 50% **50b** (9,10′ isomer), and 10% **50c** (10,10′ isomer). A bridged R unit can change, e.g., on -O-, -CH_2_-, -O-C_6_H_4_-O-, etc., also giving 3 soluble isomers, although only the one shown in Figure 19 was separated [69].

The terminal perimidine units underwent an addition reaction via electrochemical oxidation and subsequent reduction to create a multi-directional crosslinked polymeric product. The course of electropolymerization depended significantly on the isomer used, which was related to the intermediate products formed during electrooxidation: π-dimers formed in the precursor solution with stronger π–π interactions between radical cations and/or products containing π-conjugated segments: bis-isoindolinone, bis-perimidine, and protonated σ-bonded bis-perimidine, where hydrogen bonds stabilized these latter adducts and prevented their deprotonation [70].

The condensation of aromatic dianhydrides with aromatic tetramines leads to polymers containing imidazole and/or imide groups, having very high thermal resistance. The most popular polymer in this group is poly(benzimidazobenzophenanthroline) (**BBL**), which is a condensation product of 1,2,4,5-tetraaminobenzene with naphthalene-1,4,5,8-tetracarboxylic acid or dianhydride. This is a ladder polymer with the chemical name poly[(7-oxo-7.10*H*-benz[de]-imidazo [4′,5′:5.6]benzimidazo[2,1-*a*]isoquinoline-3,4:10.11-tetrayl)-10-carbonyl]. The ladder polymers consist of an uninterrupted sequence of rings with adjacent rings having two or more atoms in common (as defined by IUPAC in 1993) [71]. Due to two-dimensional geometry, this type of polymer is thus intermediate between linear and three-dimensional systems. The limited conformational freedom of conjugated ladder polymers is particularly relevant since the steric inhibition of electron delocalization is drastically reduced, but probably the structure is the random mixture of cis and trans isomers.

**Figure 20:**
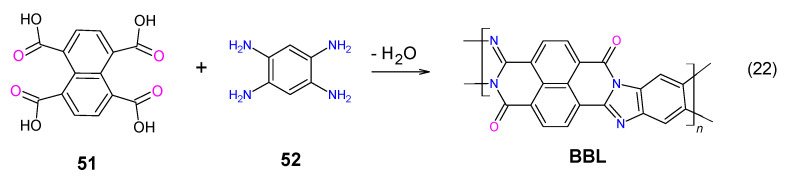
Synthesis of **BBL** polymer in polyphosphoric acid at 220 °C.

Van Deusen [72] published the first synthesis of **BBL** polymer; its preparation reaction scheme is shown in Figure 20.

The **BBL** polymer is a dark, very hard material which is sparingly soluble in concentrated sulfuric acid, polyphosphoric acid, methane- and benzene-sulfonic acids and concentrated sodium hydroxide. The extent to which complete or perfect cyclization occurred has not been established, although the analytical results indicate a very high conversion. **BBL** polymers typically show a minimal loss of weight below 500 °C in air, whereas they reach about 2–4% under nitrogen atmosphere up to 600 °C [73].

**Figure 21:**
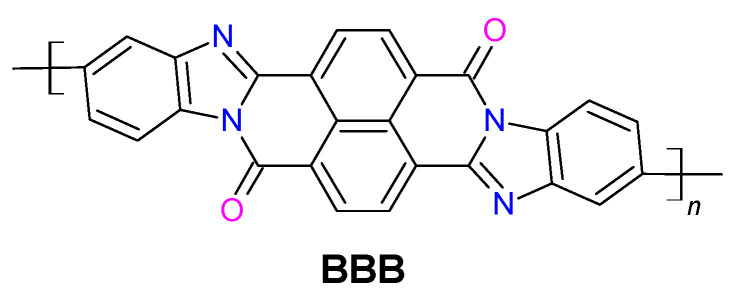
The structure of **BBB** polymer.

Another polymer of similar structure and properties is poly(6,9-dihydro-6,9-dioxobisbenzimidazo[2,1b:1′,2′-j]benzo[1mn][3,8]phenanthroline-2,13-diyl) (**BBB**), which is formed in the same condensation reaction of **51** with tetraamine. Here, 3,3′-diaminobenzidine is applied for that purpose (**53**) [72]. **BBB** is not a ladder polymer because it is composed of perinon units connected by single bonds, an example of its structure is shown in Figure 21.

Single bonds can be formed in four preferred places, thus addition to this formation of two cis and transisomers during the condensation process causes the resulting polymer to be not stereoregular but a rather complicated structure. Despite this, its interesting properties make it often useful in organic electronics [74,75,76]. The **BBL** and **BBB** polymers were resistant to ionizing radiation, and their mechanical properties fall into the following limits: the mechanical strength (on stretching), 1.05–1.54 × 10^3^ kg/cm^2^; elongation, 3 to 7%; and modulus of elasticity, 2.4–7 × 10^4^ kg/cm [77].

**Figure 22:**
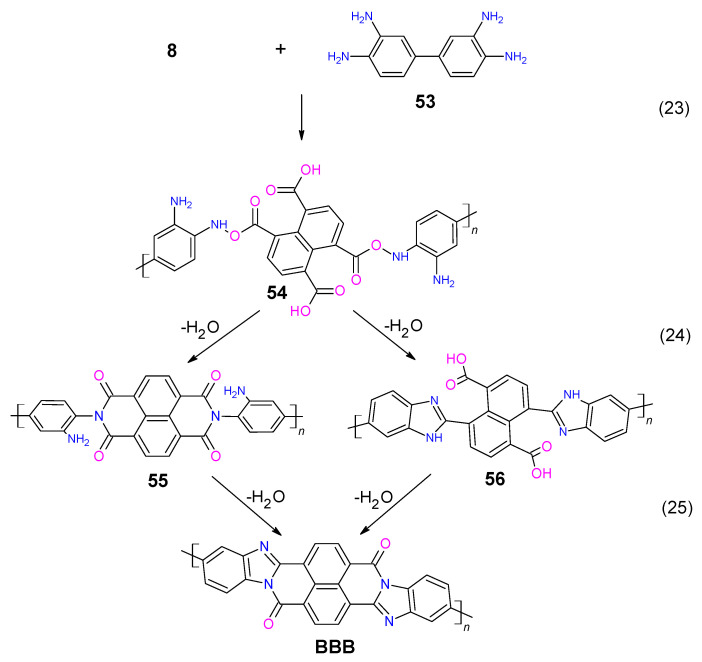
Polycondensation mechanism of **BBB** synthesis [81].

Research into model compound reactions showed, that condensation between aromatic dianhydrides **8** and tetra-amines **53** proceeds in three successive stages: formation of an amide-amino-acid, cyclisation of the latter to an aminoimide, and finally conversion of this product into an aroylenebenzimidazole polymer **BBB** [78,79,80]. The proposed mechanism of polycondensation is shown in Figure 22.

The principle of the polycondensation of tetra functional monomers used for the synthesis of **BBL** was extended to other aromatic tetraamine (1,2,4,5-tetraaminobenzene) with pyromellitic naphtha or perylene dianhydrides. Early work on the preparation and properties of such polymers was summarized by Russian researchers in the 1970s [79,82,83]. Depending on the structure of the aromatic tetramines used, completely or partially ladder poly(arylenebenzimidazole)s was obtained. In order to largely avoid the undesired formation of branched and cross-linked structures, the condensation reaction should be carried out in two stages: preliminary condensation to give single-stranded intermediates **55** and **56** at 180 °C, followed by a subsequent polymer-analogous condensation at elevated temperature (350 °C), which increases mobility of the chain fragment and promotes heterocyclic ring formation [84]. Poly(amine-amic acid) **54** is soluble in N,N-dimethylformamide (DMF) or N,N-dimethylacetamide (DMA), from which a good quality film can be cast. Thermal treatment of the solid film at 250 °C completely converts polymeric material into **BBB** polymer. Examples of components of the reaction mixtures used to prepare poly(arylene benzimidazoles) are listed in Table 1.

The polycondensation reaction can be carried out in a melt [102] or in solvents. Many different solvents were used, such as DMF, DMA, dimethylsulfoxide (DMSO), pyridine, phenol, polyphosphoric acid (PPA), and diethylene glycol dimethyl ether (Diglyme) [103]. The yield of polycondensation depends on the method of synthesis and usually was much higher in the case of the solvent method, PPA was most commonly used among them [85]. The polymers obtained from perylene-tetracarboxylic dianhydride are more resistant to thermo-oxidative degradation than polynaphthoylenebis- benzimidazoles, which is due to the stronger inter-molecular interaction of polymers containing perylene rings.

The bridged diphenyl-structured dianhydrides or tetraamine components used in synthesis of polymers disrupt continuous ladder structure, thus the so-called stepladder pyrrones could be obtained. This kind of polymer would have a lesser rigidity of the backbone and, consequently, a better processability [104,105]. It should be noted that **BBL** polymers are more sparingly soluble in sulfuric acid than the non-ladder **BBB** and may require heating to complete dissolution [103]. The **BBL** and **BBB** polymers are insulators but the fully conjugated structure allows them to convert it into conducting state upon doping [106]. However, in the case of polymer synthesis using type **59**, **60**, **66**, **67,** and **68** monomers, conjugated polymers are not obtained. They have good thermal properties [100], but they can only be redox polymers.

**Figure 23:**
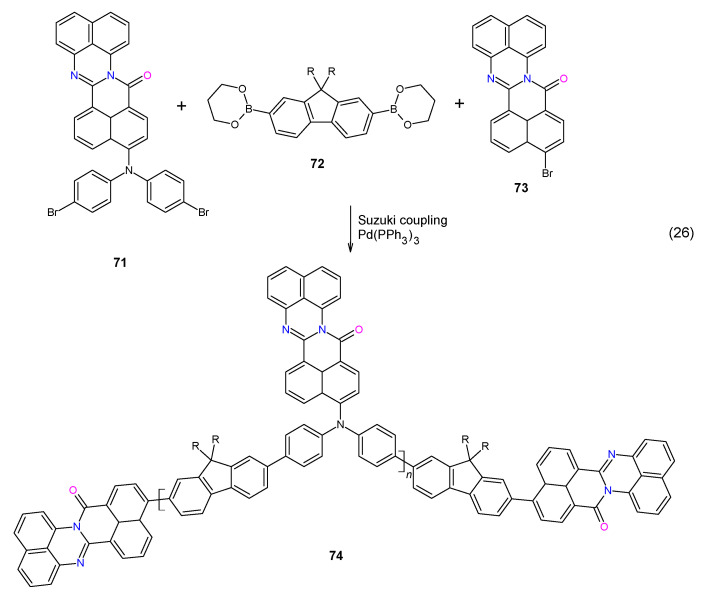
Synthesis scheme of side chain substituted by perinone derivative conjugated copolymer.

In addition to ladder polymers, conjugated polymers were also obtained in which the main skeleton was made of electron donor repeat units, while the electron acceptor derivatives of perinone were incorporated as side groups [107]. Typical synthesis is shown in Figure 23.

11-Bromo-14H-benzo[4,5]isoquino[2,1-a] perimidin-14-one (**73**) was obtained by reaction 3, where 4-bromo-1,8-naphthalic anhydride was condensed with **18**, which was then coupled with diphenylamine and further brominated to give 11-Bis (4-bromo-phenyl) amino-14H-benzo[4,5]isoquino[2,1-a]perimidin-14-one (**71**). The resulting polymer **74** was black and well dissolved in typical organic solvents, which allowed full characterization using FTIR and NMR spectroscopy. Molecular weight measurements were also made to give Mn = 9300 g/mol and Mw = 18,200 g/mol, resulting in a polydispersity factor of 1.95. In the reaction 26, the same authors obtained a similar polymer replacing diphenylamine with carbazole [108].

Photoexcitation in this type of polymer leads to charge separation, which occurs through transfer of electrons from the main chains to the side perinone group allowing to enhance charge transfer ability. The resulting polymer also absorbs light more effectively.

## 3. Properties

### 3.1. Solubility

Perinone derivatives create an important class of dyes and pigments, and their semiconductor properties combined with high stability make them potentially useful for applications in organic electronics and optoelectronics, including organic field-effect transistors (OFETs) and organic photovoltaics (OPVs). The **1** offers the advantage of a low energy gap, lower than more commonly used perylene tetracarboxylic acid diimides; moreover, its absorption may cover the whole UV/Vis absorption spectral region after modification of the molecule. The perinone is rather expensive because of the multi-step syntheses with a yield of only about 50% in the last step (due to the formation of the isomers). Because of the high price, the application of **1** is limited to areas which require high fastness properties. Due to its excellent stability against light and weathering, it is mainly used for spin-dying of polymer fibers for outdoor applications, e.g., in polyacrylonitrile fibers for awnings, tents, and canvasses. Other uses include plastic products, paints, and special printing inks. Transperinone is internationally registered in the Colour Index as “C.I. Pigment Orange 43” and “Vat Orange 7” and cisperinone is registered as “C.I. Pigment Red 194” and “Vat Red 15”, but due to its very dull shade, its value is low. The solid solution of cis and transperinone, which emerges is registered as “C.I. Vat Red 14”. It is mainly used as a vat dye, although its use has decreased [109]. It should be noted, however, that although perinone is a frequently used pigment, it is rarely used in organic electronics due to the practical lack of solubility in typical organic solvents.

The **BBL** polymer can be processed from solution into high quality films and layers, for example, by casting or by spin coating. However, in this way one is forced into the use of protic solvents that are quite aggressive and difficult to handle, for example, concentrated sulfuric acid, (PPA), or methanesulfonic acid, or, alternatively, mixtures of nitromethane with Lewis acids such as aluminum trichloride, iron trichloride, or magnesium chloride [110,111].

The monomer units of perinone (**1**) in **BBL** and **BBB** polymers interact with the acid in the simple protonation reaction:

1 + x H_2_SO_4_ ↔ 1H_x_^+x^ + x HSO4 (26)

**Figure 24:**
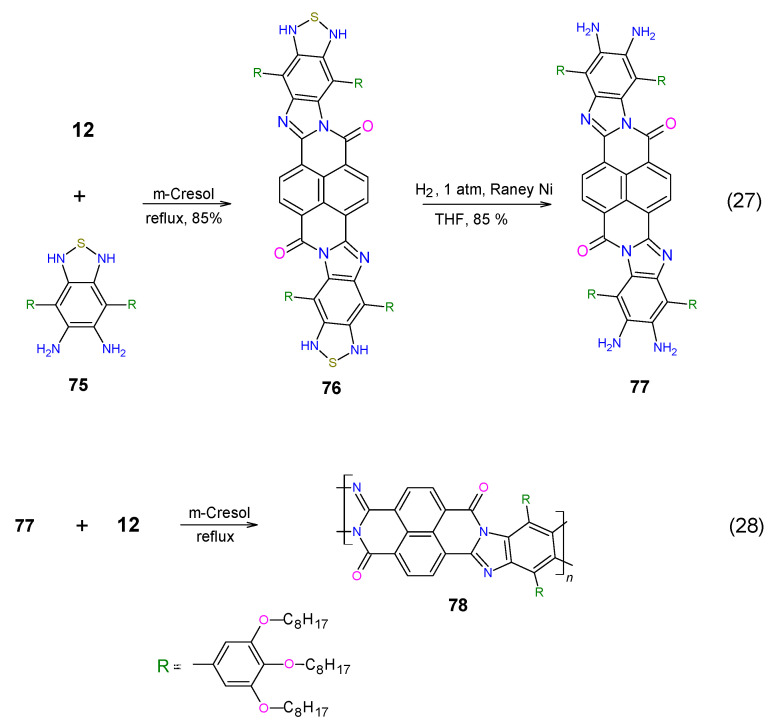
Synthetic pathway for soluble **BBL** derivative **78** [90].

The values of x for perinone scatter about a value near 2 and protonation probably involves the −N = pyrimidine nitrogen. Considering this, **BBL** or **BBB** polymers in acidic solutions can be treated as polyelectrolytes [112]. Solubility in strong acids is problematic because they may cause damage to other materials and require extensive washing and extended drying times. This problem has motivated many researchers to seek the possibility of modifying 1,2,4,5-tetraaminobenzene by substituting it with volumetric substituents in free places. One example of the synthesis of soluble ladder polymers of perinone structure was presented by Wang et al. [90,113]. Their strategy consisted of a two-step reaction shown in Figure 24.

In the first stage, well-defined naphthalene-containing double-stranded heterocyclic tetraamines **77** as the building blocks was synthesized, which in turn is employed to obtain **BBL**-like derivatives by further condensation with aromatic dianhydrides (reaction 28). The method seems to be very flexible because it allows the synthesis of copolymers; moreover, it is possible to use different aromatic dianhydrides in the first stage and others in the second. Large twisted aryl- and long alkyl-pendent groups were introduced at the lateral positions of the building blocks to impart the solubility to the final polymer. Finally, these **BBL**-like polymers are readily soluble in common organic solvents, absorb intensely in the visible and near-infrared region, and show high electron affinity and moderate mobility, which resulted in a conductivity lower by 3 orders of magnitude compared with that of **BBL** [90,113].

Briseno et al. [114] prepared large quantities of solvent-dispersible **BBL** nanobelts in environmentally benign solvents such as methanol or water by precisely controlling the solution-phase self-assembly of the **BBL** polymer. The molecular packing of polymer chains in nanobelts reveals a self-assembled motif unlike the traditional π-stacking common to currently known small organic molecules or polymer semiconductors [115,116]. The **BBL** nanobelts are dispersible in a large number of solvents including environmentally benign solvents such as methanol and water. This dispersions was used for fabricating organic transistors from environmentally benign alcoholic or aqueous solvents under ambient conditions and they have shown stable and reproducible performance in air for well over 6 months. The stability of the **BBL** nanobelt electronic devices arise from the favorable electronic and molecular structure of the polymer as well as the morphology of the oriented polymer chains within the nanobelts [114].

**Figure 25:**
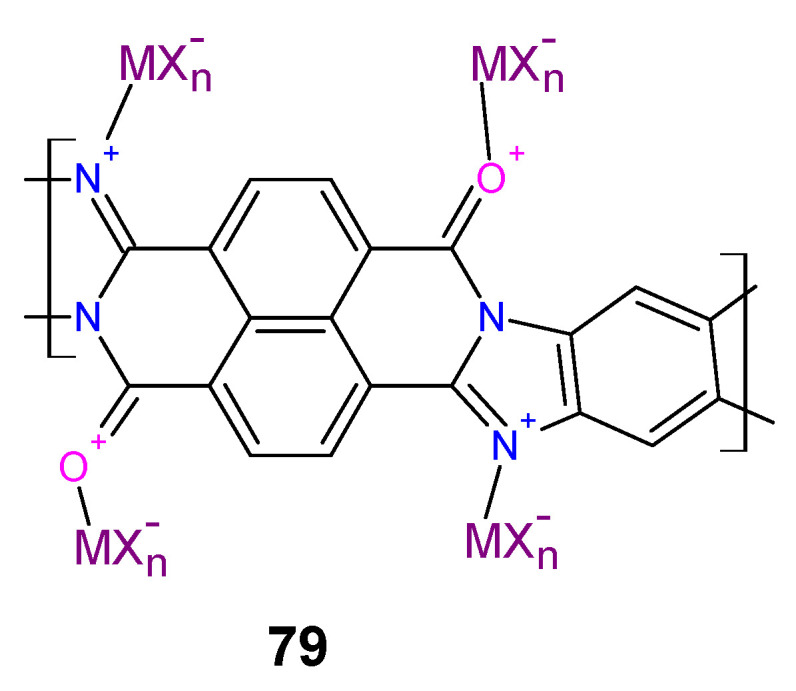
Structures of the soluble Lewis acid (MX_n_) complexes of rigid-chain **BBL** polymer.

The known solubility of many rigid-chain polymers in strong protonic acids such as methanesulphonic acid (MSA) and camphor sulphonic acid (CSA) has been established to be due to protonation of the polymer chain, thereby forming polyelectrolytes and consequently reduction of intermolecular attractions and chain stiffness [117]. It is possible to dissolve **BBL** in aprotic organic solvents, such as nitroalkanes and nitrobenzene, containing metal halide Lewis acids (MX_n_) as the complexing agents [111]. The effectiveness of the Lewis acids in promoting solubilization of **BBL** in nitromethane was in decreasing order: GaCl_3_ > AlCl_3_ > FeCl_3_ > SbCl5 > SbCl_3_. The main factor that determined solubility of the rigid-chain polymers was the solvation properties of the organic solvent/Lewis acid system and the stoichiometric Lewis acid MX_n_/**BBL** (repeating unit) ratio equal to 4:1. Structures of the soluble Lewis acid (MXn) complexes of BBL polymers **79** is shown in Figure 25.

The reaction of Lewis acid with **BBL** occurs site-specifically at the heteroatom donor sites on the macromolecule. The resulting product of the reaction is an electron donor-acceptor (EDA) complex in which a localized coordinated covalent bond is formed between the metal atom of MX_n_ and the donor heteroatoms oxygen and nitrogen. The EDA complex is a neutral molecular adduct compound but possesses highly polarized properties relative to the pristine materials. This complex require of a polar organic solvent (high dielectric constant) and a low donor number for solvation [111,118]. The main disadvantage of using such solutions is the difficulty in removing Lewis acid from the **BBL** polymer film.

Janietz and Sainova [119] prepared water dispersions of **BBL** with controllable particle size in the range between 50 and 150 nm, which was used to form layers by spin-coating or drop-casting. The resultant films exhibited an ambipolar charge carrier, which presumably was related to the presence of mobile ions. It was assumed that during the preparation of the aqueous dispersion, a very small amount of the MSA becomes strongly adhered to the **BBL** backbone.

**Figure 26:**
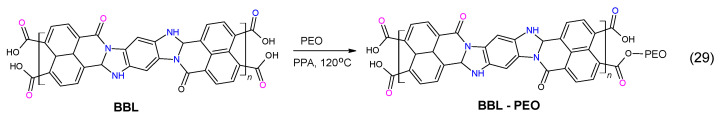
Preparation of **BBL–PEO** block copolymer in polyphosphoric acid (PPA) solution.

Hirvonen et al. [120,121] prepared amphiphilic **BBL**s with poly(ethylene oxide) attached to the chain ends. The reaction is presented in Figure 26.

After the preparation of **BBL** according the reaction (22), the temperature of the reaction mixture was decreased to 120 °C and poly(ethylene oxide) monomethylether (PEO-MME) was added in a minimum of four times molar excess with respect to the theoretical number of carboxylic acid end groups in **BBL**. BBL–PEO copolymer was recovered as a black paste and stored as suspensions. The suspensions were processed into visibly transparent blue dispersions and used for physicochemical characterization. **BBL–PEO** composite is less thermally resistive compared with pure **BBL** polymer because pure PEO thermally decomposes in quite a narrow region of 320–440 °C, but these dispersions can be used to prepare films using spin coating or drop casting. The obtained films were uniform with film thicknesses varying between 20 and 30 nm [120]. Detailed study showed that **BBL–PEO** dispersions form aggregates and form gels with increased concentration. The strongest gels are formed from **BBL–PEO**s with short PEO chains [121,122].

### 3.2. Spectroscopic Properties

**Figure 27:**
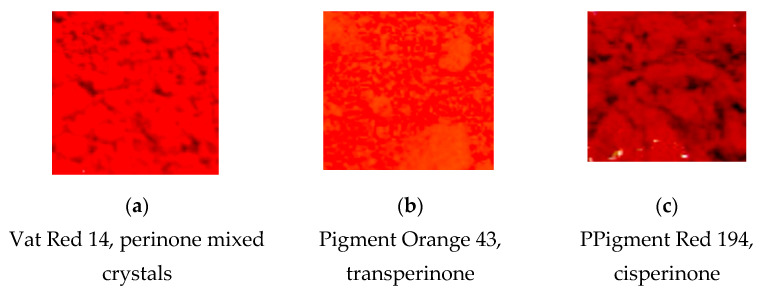
Colors of **1** isomers: (**a**) solid solution of cis and trans isomers; (**b**) transperinone; and (**c**) cisperinone [34].

The color of both perinone **1** isomers as well as of the solid solution are shown in Figure 27. They form insoluble fine powders in shades ranging from orange to deep red and demonstrate very high heat stability (melting points far above 673 K) and are very lightfast and weatherfast [123]. Due to this and quite a high price they are mainly used for spin-dying of polymer fibers for outdoor applications, e.g., in polyacrylonitrile fibers for awnings, tents, and canvasses. Other uses include plastic products, paints, and special printing inks.

An important reason for interest of perinone type pigments is their pronounced crystallochromy, i.e., their color strongly depends on the crystal structure. Both isomers of perinone have similar crystal structure, but the molecular arrangement and the direction of the transition dipoles are slightly different. In both compounds, an additional absorption band appears at longer wavelengths upon crystallization due to excitonic interactions between transition dipoles. These bands play an important role in the determination of the shade of transperinone and cisperinone in the solid state [124,125,126]. The solid solution consisting of both perinone isomers shows a combined positional and orientational disorder. Lattice-energy minimizations with DFT-D methods reveal that the solid solution contains both orientations of the cisisomer, but only one orientation for transmolecules. All models containing any transperinone molecules in the second hypothetical orientation are energetically unfavorable, regardless of the arrangement of the other molecules [109].

**Figure 28:**
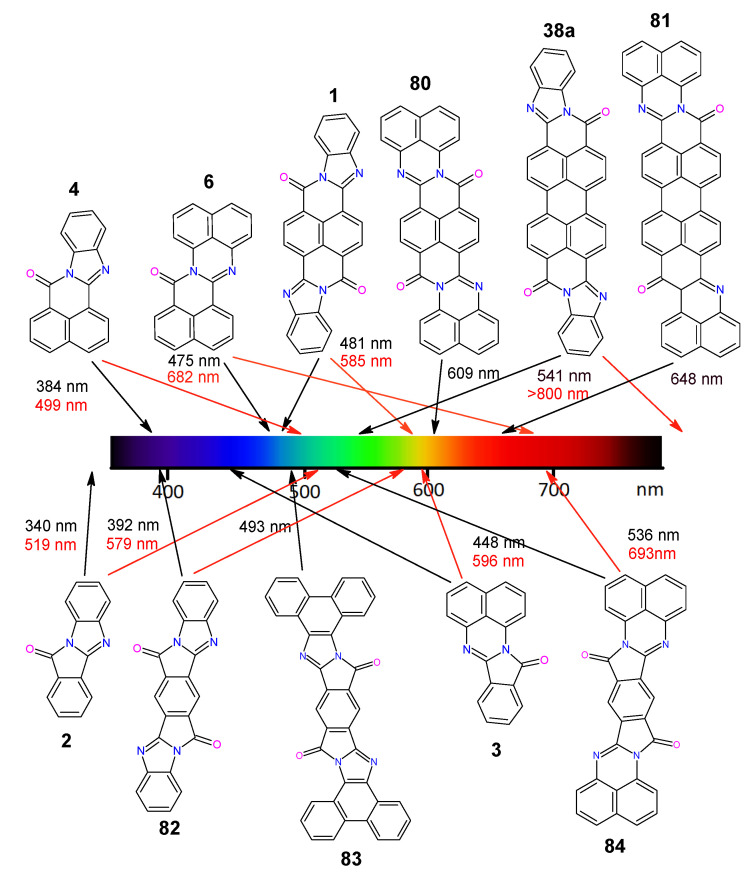
Some examples of perinone derivatives and their maxima of absorption in solution (marked in black) and in solid phase (marked in red). Data selected from work [129].

It is well known that an increase in the size of the π-conjugation causes a shift toward a longer absorption wavelength (bathochromic shift) [127,128]. The selection of appropriate aromatic carboxylates and diamines allows the conjugation length of the obtained perinone derivatives to be to easily controlled. It should be added, however, that in the case of solid state materials, the color largely depends on the structure of the perinone derivatives because the molecular structure controls the intermolecular interaction in the solid state and determines the crystal packing. In Figure 28, examples of perinone derivatives with different conjugation length and their maxima of absorption in solution (marked with black arrow) and maxima of luminescence peaks (marked with red arrow) are listed.

The spectra of perinones substituted with electron-withdrawing groups were blueshifted, and with electron-donating groups redshifted. A significant bathochromic shift of the absorption of perinone molecule was obtained by using in synthesis 1,8-naphthylediamine which form six-membered amidine moieties **3**, **6**, **80**, **81**, and **84**. The compounds with electron-withdrawing groups display high molar extinction coefficients; conversely, the compounds with electron-donating groups display low extinction coefficients. For example, extinction coefficients of unsubstituted **4** was 13,000 M^−1^cm^−1^ while substituted by -NO_2_ group showed 26,000 M^−1^cm^−1^ and by –CN group only 8000 M^−1^cm^−1^ [129]. The majority of perinone compounds are fluorescent. The emission maxima show spectral shifts corresponding to their UV/Vis absorption maxima. The Stokes shifts remained substantial across the series, frequently exceeding 100 nm. The molecules substituted with electron-donating groups exhibited relatively lower quantum yields. On the other hand, perinones with electron-withdrawing groups, such as the cyano group, generally resulted in higher quantum yields (70–80%) and may have potential as blue emitters in organic electronics [129].

Compared with their oxo-analogues, thioxo-perinones **3S** and **4S** have unique photochemical and photophysical characteristics including a progressively bathochromic absorption shift, increased molar absorption coefficients, dramatically enhanced intersystem crossing efficiency, which could be utilizing in the design of novel fluorescent probes based on the reaction of thiocarbonyl, highly potent DNA photocleavers, and promising photosensitizers in cancer therapy [40].

UV–vis-spectrum of neutral **BBL** in solution recorded in methanesulphonic acid (MSA) shows absorption maximum for π–π*-transition at 540 nm and the onset of the absorption at 574 nm. BBL formed a deep blue dispersion in water with two absorptions maxima located at 580 nm (π–π*) and 354 nm (n–π*) [120]. The color of the film depends significantly on the method of its preparation because it is very often difficult to remove the substances used to ensure the solubility of **BBL**. The pristine **BBL** solid film exhibits a sharp increase in absorption above 690 nm with an absorbance peak located at 565 nm. This absorption peak is associated with the extended state π–π* band transition in highly conjugated ladder polymer. The optical band gap for this polymer in solid state is about 1.8 eV.

The absorption maximum was hypsochromically shifted to 550 nm, in the film of **BBL**/poly(styrene-sulfonic acid) (PSSA) complexes, but absorption edge remained unchanged [104]. The absorption spectra of **BBB** was similar to **BBL** in solution and in solid film, however the absorption maximum of **BBB** complexes with Lewis acids was hypsochromically-shifted by 25–29 nm relative to the BBL complexes. The optical band gap for **BBB** in solid state was slightly larger, it′s about 1.81 eV. This is due to the fact that **BBL** is known to be highly crystalline [130] whereas random-coil **BBB** molecules form amorphous [131,132] polymer films.

### 3.3. Conductivity

The **BBL** and **BBB** films are insulators (σ = 10^−12^ Ω^−1^cm^−1^) but thermally annealed at ca. 500 °C, 24 h in vacuum, turned out semiconductive (σ ≈ 10^−6^ Ω^−1^cm^−1^) without appreciable changes in dimension and appearance, while for annealing temperature T > 900 K, the conductivity rises to σ = 3 Ω^−1^cm^−1^ [75]. This dramatic change in the in-plane conductivity, by 8 orders of magnitude, was probably associated with a "phase transition" to a rigid cross-linked network. This new highly conducting phase could be formed by the condensation of "defects" or "free radicals" between adjacent rigid rod polymers. The rational explanations for this phenomenon was based on increased structural perfection of the ladder structure or thermal activation to generate free radicals.

Halogens such as iodine and bromine had a negligible doping effect on **BBL** or **BBB** polymers, which indicates that they are insufficiently strong oxidizing agents [133]. Neutral films could be doped with Lewis acid or protonic acids. After doping, the original golden color of the films became green, dark green, or green-black, depending on the extent of doping. A significant effect was observed with AsF_5_ doping at room temperature for 2 days (σ = 10^−3^ Ω^−1^cm^−1^), but after doping with sulfuric acid or with SO_3_ conductivity increased even more (σ = 1 × 10^−2^ and σ = 2 Ω^−1^cm^−1^ respectively). After treatment, the doped films remained flexible but the film thickness increased 20–25% and the weight increase was nearly 200%, corresponding to the molar ratio of the H_2_SO_4_ dopant with respect to the repeating unit in the **BBL** ranging from 6:1 to 8:1. The **BBB** polymer behaves similarly, obtaining a conductivity of σ = 1 and 1 × 10^−3^ Ω^−1^cm^−1^, respectively, for doping SO_3_ or sulfuric acid [134]. The doping of potassium naphthalide was most effective and conductivity of both polymers exceeded the value σ = 1 Ω^−1^cm^−1^, corresponding to a 12-order-of-magnitude increase in the conductivity.

Jenekhe et al. [104] protonated **BBL** and **BBB** polymers with poly(styrenesulfonic acid) (PSA). They observed that the transition from insulator to electronic conductor (2 S/cm) occurs when neutral **BBL** or **BBB** was about 70–100 mol% singly protonated. This transition was accompanied by a large shift in the formal reduction potential from −0.85 and −0.69 V for neutral **BBL** and **BBB,** to 0.35 and 0.33 V versus SCE, respectively, for the protonated conducting forms. Although the HOMO–LUMO optical band gap remains unchanged at 1.8 eV. These results showed that the electronic structures of conjugated ladder polymer **BBL** and its semi-ladder analogue **BBB** undergo a large change upon protonic acid doping. The detailed theoretical study of **BBL** electronic structure upon doping was given by a Swedish group [135]. They found that the electronic structure of the n-doped polymers is qualitatively different from that in traditional predictions. For the case of the polaron state, the electronic structure of **BBL** was qualitatively similar to those of n-doped Polythiophenes and polyparaphenylenes calculated using the DFT approach. The ground state of two electrons in a chain corresponded to a triplet polaron pair, which was in stark contrast to a commonly accepted picture where two electrons formed the bipolarons. With further increase in the reduction level, the total spin decreased until it became 0 for the reduction level equal to 200%. The calculations were correlated with experimental results, which showed changes in the number of spins during electrochemical doping [136].

**Figure 29:**
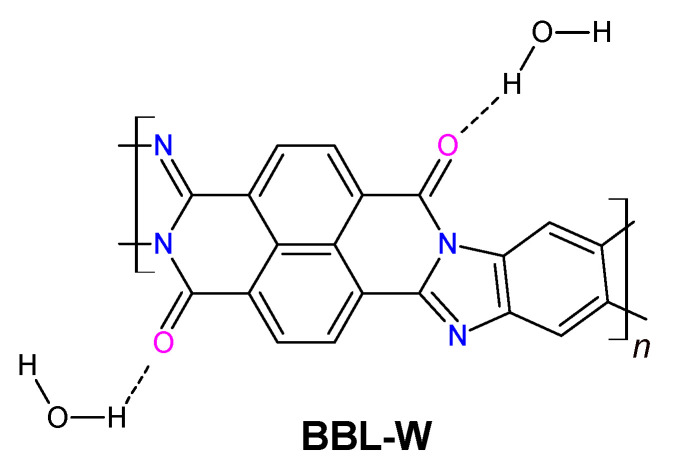
Chemical structure of **BBL** showing possible water attachment to the chain through hydrogen bonding to the carbonyl oxygen (**BBL-W**) [137].

Antoniadis et al. [137] showed that the dark conductivity and the dielectric constant of thin films of the **BBL** polymer exhibit large sensitivity to the relative humidity. The authors did not prove the acceptable mechanism of this process; however, the measurements carried out suggested that changes in conductivity were associated with the formation of hydrogen bonds between carbonyl oxygen and water, forming a **BBL-W** type polymer, as shown in Figure 29.

Thermogravimetric measurements show that only 2 wt % moisture was absorbed in the **BBL** film, thus only about 20% of the carbonyl oxygen sites in the polymer film have absorbed water molecules, which might suggest that only molecules located close to the surface of the thin film were affected by humidity [137].

The electron-donor doping of the **BBL** polymer film with metallic potassium was reversible and conductivities increased to σ = 2 × 10^−1^ Ω^−1^cm^−1^ value. A conventional donor doping with potassium naphthalide in THF solution at room temperature allowed to achieve even greater conductivities value (σ = 1 Ω^−1^cm^−1^). In the case of **BBB**, the same doping allows to achieve a slightly lower conductivity of 1 × 10^−1^ Ω^−1^cm^−1^. The donor-doped films were rather sensitive to air exposure compared with the acid-doped one but the deterioration rate was much slower compared with other conjugated polymers. An enormous increase in thickness and weight of the doped films was observed which was explained by the planar layered structure of ladder polymer [134].

Another way to increase the conductivity of semiconductor materials, including organic conjugated polymers, is ion implantation which allow introduction of controlled amounts of impurities into materials to modify their electronic properties. This method was also used for **BBL** [130,138]. Polymer conductivity increases with increasing radiation dose reaching saturation state, which depends on implanted ion and beam current density. Table 2 shows the obtained conductivity of **BBL** films as a function of beam current density and selected ions (J_b_).

The gold-yellow pristine **BBL** films gradually turned black and then silver-gray with metallic luster as the concentration of implanted impurities increased. Correspondingly, the optical reflectivity increased with dose. The excellent mechanical properties of the films, including flexibility, were retained after implantation to doses of 4.0 × 10^16^/cm^2^ or higher. This suggests that a significant portion of the original **BBL** sheet structure is retained after implantation. The Authors proposed possible intermediate structure resulting from partial carbonization by implantation, in which **BBL** monomers were deprived of oxygen atoms. The optical, infrared, ESR, and electrical data suggest that the rigid ladder BBL polymer system maintain some of its structural integrity even after moderately heavy exposures to high-energy ions. The irradiated films exhibit electrical conductivity that was stable at 250 °C in air for hundreds of hours [138].

The **BBL** polymer has been studied as n-type material for thermoelectric devices. For this application, it has been doped either with the help of the strong reducing agent tetrakis (dimethylamino)ethylene (TDAE) or via base doping with benzimidazole derivatives such as 4-(2,3-dihydro-1,3-dimethyl-1H-benzimidazol-2-yl)-N,N-dimethyl-benzenamine (N-DMBI). Reduction of **BBL** with TDAE, for instance, gives rise to an electrical conductivity of about 1 S/cm and a Seebeck coefficient of -60 μV K^−1^ (α^2^σ ~ 0.4 μW m^−1^K^−2^) [139,140].

### 3.4. Electrochemical Properties

The electrochemical properties of products were frequently used to determine the HOMO–LUMO levels of materials from their oxidation and reduction potentials. Cyclic voltammetry (CV) and differential pulse voltammetry (DPV) are most often used for these determinations. The precise knowledge of the HOMO–LUMO levels is crucial when the conjugated organic materials are used in optoelectronic devices such as light emitting diodes (LEDs) or photovoltaic cells. In fact, CV or DPV measurement allows to determine the ionization potential during oxidation and electron affinity potential during reduction though only under certain conditions electrochemical results can be used to obtain the HOMO–LUMO value [141,142]. All small molecular derivatives of perinone are electroactive in cathodic region which allow to obtain electron affinity potential and the LUMO level. Although some of the compounds with a larger conjugated core also showed a electro-oxidation process. When only the reduction or oxidation process can be registered, an optical band gap can be used to determine the missing potential. Table 3 presents examples of oxidation (E_ox_) and reduction (E_red_) potentials as well as HOMO and LUMO energy levels of selected perinone derivatives.

The compounds **25–27** underwent multiple reversible or pseudo-reversible reductions upon cathodic scan. No oxidation waves were observed during anodic scan except for those of the acyclic trimers. Each imide subunit of these molecules accepted two electrons, with the tetramer **27** being a remarkably eight-electron acceptor [53].

The electrochemical activity of electrically conductive polymers is an important indication of their oxidative or reductive transformation into a conducting state (i.e., doping). Due to the presence of different heterocycles in **BBL** structure, electroactivity of polymer films on electrodes depends strongly on the solution pH and also on the particular electrolyte ions present in the electrolyte [146]. Regardless of the pH value, **BBL** film is electroactive showing a two-step reduction process however, the location and height of the peaks depend on the acidity of the solution. Three regions of pH can be distinguished:

Region 1. Above pH 7 (up to 12), the voltammetric pattern changes very little with pH and the voltammogram shows two well-defined waves.

Region 2. Between pH 7 and 4, the voltammogram shows a poorly resolved more negative wave and the main wave shifts toward more positive potentials.

Region 3. At pH 4, only a single wave is evident but with a substantial peak splitting. At lower pH, the peak splitting diminishes and the wave shifts positively and divides into two waves.

More detailed study of Wilbourn Murray [147] showed that **BBL** material can exhibit the behavior of redox polymers and classical conducting polymers; moreover, that transformation from one characteristic to another depends on the polymer′s environment. Using in situ UV–Vis, and Raman and FTIR Spectro electrochemistry, Sariciftci et al. [148] found four distinct and spectroscopically well-resolved redox processes during electrochemical reduction (n-doping) of **BBL** film, involving five redox states with different conductivities. In correlation with the in situ electrical conductivity data [147], four reactions of doping process were proposed:

- **BBL**(I, insulator) + 0.25e^−^ ⇄ **BBL**^0.25−^ (II, conductor)        (30)
- **BBL**^0.25−^ (II, conductor) + 0.25e^−^ ⇄ **BBL**^0.50−^ (III insulator)      (31)
- **BBL**^0.50−^ (III, insulator) + 0.35e^−^ ⇄ **BBL**^0.85−^ (IV, conductor)      (32)
- **BBL**^0.85−^ (IV, conductor) + 0.15e^−^ ⇄ **BBL**^−^ (V, insulator)        (33)

The overall absolute charge during the reduction process was determined with coulometric assay to be equivalent to a consumption of one electron per **BBL** monomer unit, although the exact determination of the charges involved in the individual reduction steps is hindered by the overlapping peaks in the CV; therefore, the load values given in equations A–D should be treated as approximate [148]. The electron spin resonance (ESR) spectroelectrochemistry and in situ conductivity experiments on **BBL** samples allow to correlate unpaired electrons with the number of electrons per repeat unit (ERU), as functions of electrochemical doping potential [136]. No mobile electrons were observed in the neutral state. At doping levels lower than 0.5 ERU, conductivity and electron spin concentration increase together, suggesting that the conducting species have spin 1/2. When the electron concentration reaches a level of 0.5 ERU, conductivity reaches a maximum, even though the electron spin concentration has not yet maximized. A maximum conductivity at this doping level is consistent with a simple redox hopping of electrons from singly occupied to empty sites suggested in [147] for dry condition of **BBL** doping. **BBL** polymer possess electrochromic properties. The spectra taken at potentials that span the voltammetric waves showed an increase and decrease in absorbance at 524 and 583 nm, respectively, and two not very well-defined isosbestic points at 375 and 550 nm. For the most reduced form, the maximum peak is located at 503 nm [149].

Another way to improve the electrochemical properties of **BBL** has been shown by Irvin et al. [150] They prepared ternary solution of **BBL**, 1-ethyl-3-methylimidazolium bis(trifluoromethylsulfonyl)imide (EMIBTI) and trifluoroacetic acid (MSA) which was homogeneous at 90 °C. Using this solution, the polymer film was cast onto an appropriate substrate. The current response was one order of magnitude higher compared with the **BBL** film cast without EMIBTI. Scanning electron micrographs showed greatly increased surface roughness and porosity of the film cast from ternary solution compared with the case of simple **BBL**/TFA solution.

**Figure 30:**
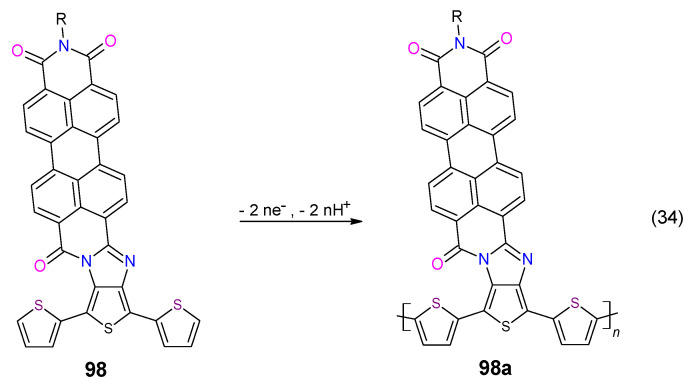
Scheme of electrochemical polymerization of terthophene perylene-amidine-imide derivatives 98 [64].

The oligothiophene derivatives covalently linked to perylenetetracarboxylic acid diimide moieties have shown promising results as an active layer in organic photovoltaic devices. Bauerle and coll. [64] synthesized a molecule having perylenemonoimide moieties directly fused and conjugated to the terthiophene monomer using imidazo cycle. This molecule could be polymerized electrochemically giving an ambipolar polymer. Polymerization reaction is shown in Figure 30.

The obtained polymers exhibited homogeneous morphologies and strongly adhered to the surface of the electrode. The cyclic voltammetry of the polymer film clearly exhibits the characteristic broad redox wave of a polythiophene backbone with the peak current located at 0.52 V vs. Fc/Fc^+^. In the negative potential regime, one stable nonsymmetrical redox couple responsible for the transfer of two electrons was observable. The cyclic voltammetry of perylenemonoimide moieties showed typical redox type behavior. Regardless of the thickness of the film on the electrode, peryleneamidine monoimide units were electroactive, which indicate a good delocalization of charges on the fully conjugated moieties, as well for the p- and n-doping [64]. It should be noted that in this case, the perinone group was not located in the main backbone of the conjugated polymer.

**Figure 31:**
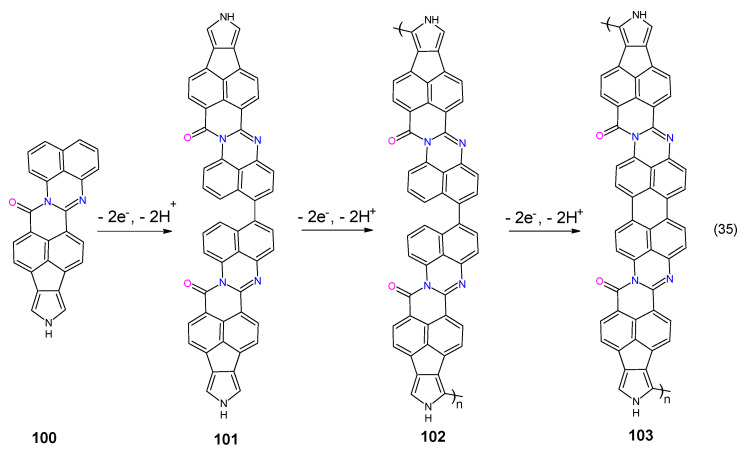
Electrochemical polymerization reactions of pyrrolo[3″,4″:2′,3′]indeno [6′,7′,1′:4,5,6]isoquinolino[2,1-a]perimidin-6(2H)-one (**100**) in dichloromethane solution.

Stępien et al. [151] obtained a hybrid structure of nonsymmetric perinone consisting of naphthalenemonoimide and pyrrole, which was electrochemically polymerized [151] as shown in Figure 31.

**Figure 32:**
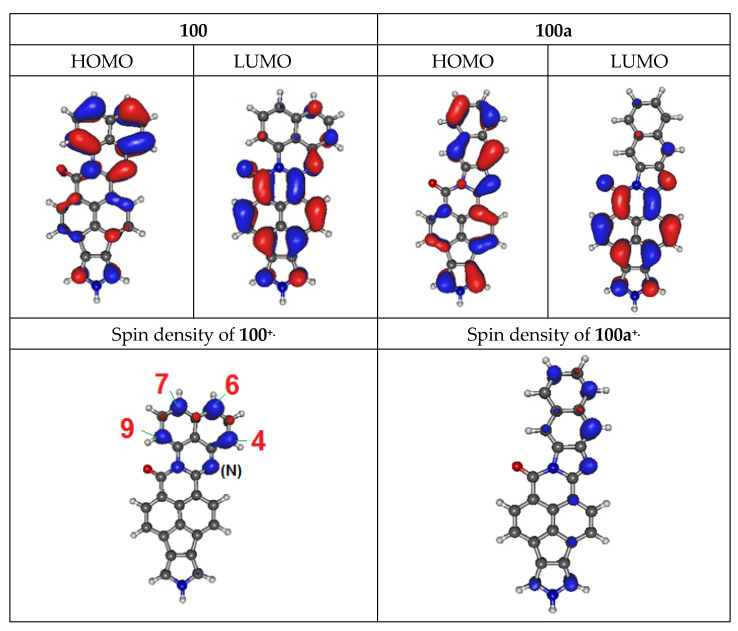
The HOMO and LUMO orbitals for molecules **100** and **100a**.

This planar monomer (**100**) containing two segments, pyrrole (donor) and perimidine (acceptor) blocks, was capable of two-direction coupling and forming a crosslinked, fully conjugated polymer. The surprising process in this reaction scheme is that the first step was the coupling of two molecules in the electron-withdrawing part and not in the electron-donor part, as is usually the case with donor-acceptor monomers. It should be noted that this reaction does not occur for naphtho [2′,3′:4,5] imidazo [2,1-a] pyrrolo [3′,4′:2,3] indeno [6,7,1-def] isoquinolin-6 (2H)–one (**100a**), in which the five-membered imidazole ring is presented, but only for the six-membered perimidine derivatives. One would expect that the HOMO orbital will be located on the acceptor part of the molecule, i.e., pyrrole ring, which is characterized by low ionization potential, but DFT calculations shoved its location onto the perimidine part of the molecule as shown in Figure 32.

The LUMO orbital of monomer **100** was localized on the isoquinolino[2,1-a]perimidin unit and the adjacent ring, while in the **100a** molecule it was delocalized virtually all over the molecule. The highest spin density in radicalcation forms of monomer **100** was localized on the perimidine unit at positions 4, 6, 7, and 9, and at nitrogen atoms, while again in the **100a** it was delocalized all over the molecule. The lowest oxidation potential (and the HOMO energy) of **100** was +0.38 V (−5.40 eV) and a stable solid product was deposited on the electrode. In the case of the reference compound **100a**, the potential of the first oxidation stage shifted to a much higher potential of +0.72 V without any film deposition. The polymer film obtained from monomer **100** possesses bipolar character with a very large (more than 1.5 V) and very stable conductive n-doping region, characterized by the typical shape of a classical conducting polymer. The onset of reduction was very low, located at above −0.2 V, but p- and n-doping ranges were not clearly separated, suggesting that no stable neutral state exists, or by the fact that the electrochemical band gap was very narrow, below 0.2 V. Therefore, it must be said that this is the first n-conductive polymer obtained electrochemically during a typical electro-oxidation process, in which the electron-withdrawing part of the molecule takes part, significantly increasing the length of the conjugation. [151].

### 3.5. Organic Electronic Applications

The perinone molecules and **BBL** or **BBB** polymers are organic semiconductors with very high photo- and thermo-stability, which is potentially useful for applications in organic electronics. Development of perinone derivatives has been limited so far by their insolubility in common organic solvents, hindering a fine-tuning and a shift of their absorption to longer absorption wavelengths. It should be emphasized that perinone (**3**) was used in the first organic thin-film solar cell with the power conversion efficiency of 0.95%, which was a major breakthrough in organic solar cells [152]. In 2000, this efficiency was more than doubled by incorporating an exciton-blocking layer, inserted between the photoactive organic layers and the metal cathode in the solar cell [153]. The performance of organic electronic devices are tightly related to the microstructure of the molecular active layer and are affected by factors such as material solubility [154], donor/acceptor ratio [155], thermal/solvent annealing [156], charge separation efficiency [157], aggregations of active molecules [158,159], and use of additives [160]. The molecules with large planar structures tend to form strong aggregation and excessively large domains (up to ≈1 µm) in bulk heterojunction solar cells which is detrimental to the organic solar cells performance. Reducing the intermolecular aggregation and the domain size can be achieved by using molecules with twisted structures [161]. However, the twisted structures generally lead to low crystallinity and poor electron transportability [162]. High electron mobility is one of the main requirements for organic materials in organic transistors and other organic devices because they support large electronic currents and yield large current modulation. This is why a great amount of effort has been put into finding new materials with high charge mobility and, in particular, high electron mobility.

The perinones generally work as n-type semiconductors as they are able to form stable radical anions, and thus they were used in the first thin-film solar cell [152]. The basic physicochemical properties of organic semiconductors include fluorescence quantum yields (Φ_F_), lifetime (τ), band gap (E_g_), and charge mobility. These parameters are collected in Table 4.

The measured values of mobilities depend on many factors, such as configuration of the measuring system, measurement in neutral or atmospheric conditions, and type of R substituent in the molecule and temperature treatment. For most perinone derivatives, mobility values were in the order of 10^−3^–10^−5^ cm^2^V^−1^s^−1^. Noteworthy are 2 compounds, **95** and **97**, for which mobility increases to 0.35 and 0.15 cm^2^V^−1^s^−1^, respectively. Much lower mobilities, in the order of 0.15 to 10^−4^ cm^2^V^−1^s^−1^ are recorded for the similar **96** and **3** molecules, which differ from planar **95** and **97** molecules by the presence of terthiophene moiety. In this case, the oligothiophene backbone decreases the electrical performance of both molecules due to skeletal distortions [95]. It should be noted that different alkyl substituents tune mobilities and solubility of molecules but have negligible effects on redox properties. Film morphology and particle orientation are other important factors that have a major impact on charge mobility in a layer. The optimal orientation for efficient charge transport between the source and drain electrodes depends on which cofacial π-conjugated planes of perinones are aligned perpendicular to the dielectric substrate surface.

Pristine **BBL** has an optically determined semiconductor band gap of 1.8 eV but an insulating dc conductivity (10^−14^ to 10^−12^ S/cm). It displays large nonlinear optical properties [165], and good photoconductivity [166,167,168]. Field-effect mobility of electrons was found to be as high as 5 × 10^−4^ cm^2^V^−1^s^−1^ in **BBL** thin-film transistors fabricated by spin coating [169], whereas it is 7 × 10^−4^ cm^2^V^−1^s^−1^ in transistors fabricated using a nanobelt network [114,170]. The field-effect mobility of electrons in **BBL** thin films was found to vary by 2 orders of magnitude depending on the solution thin-film processing method used. The highly efficient π–π interchain stacking of this ladder polymer, is in the origin of higher electron mobilities and could be improved in thin-film processing, control of the thin-film morphology, and purity of the material [171]. The influence of processing conditions on n-channel field effect transistor (FET) characteristics of **BBL** films is presented in Table 5.

The intrinsic mobility of electrons in **BBL** thin films depends strongly on solution processing methods. In the case of the **BBL** film prepared using Lewis acids, the mobilities were low, most likely due to the amorphous nature of the materials. In the case of normal operating conditions, the conjugated polymer at the semiconductor/electrode interface is oxidized or reduced during charge injection. The charge transport is realized by the ″hopping″ of polarons or bipolarons from one molecule to another. The presence of ions in the **BBL** film allow for electrochemical doping. As a result, a heavily doped channel is formed between the drain and source electrodes, which results in high drain-source currents. The presence of both positive and negative mobile ions in the **BBL** film enables field induced n- and p-doping. As a result, the ambipolar gate-modulated device was obtained [172].

The performance of the solution-processed n-channel polymer OTFTs in terms of electron mobility and electrical stability can be significantly enhanced by applying an appropriate polymer dielectric buffer layer. This layer not only enhances field-effect mobility but also stabilizes the electrical characteristics [176]; however, the electron mobility exponentially decays as the dielectric constant of the buffer layer increases [175]. The **BBL** and **BBB** comparison shows that higher mobility values are obtained for ladder polymer. The enhanced field-effect electron mobility and better overall transistor performance in the **BBL** is consistent with its generally greater electron delocalization, smaller optical bandgap, planar chain structure, closer intermolecular π-stacking interactions, and enhanced crystallinity [170,177]. The DFT calculations indicate that both polymers have the ground-state LUMO clouds stretched along the backbone, but the orbitals of **BBL** are more delocalized, whereas in the **BBB** regions a very small electron density exists due to the presence of a single bond in the polymer structure [174]. The major difference between the two polymers was morphology; **BBL** films, spin coated or cast from MSA solutions were semi crystalline, whereas **BBB** films were completely amorphous [74].

The binary blends of donor and acceptor conjugated-polymer semiconductors, **BBL**/PTQ and **BBL**/POT, showed a phase-separated morphology with domains on length scales of 50 to 300 nm. Ambipolar charge transport was observed in the **BBL**/PTQ blends with more than 90 wt % PTQ. The absence of ambipolar charge transport for most of the binary blend composition shows that the thin-film morphology and the phase-separation dynamics are critical for observing ambipolar charge transport in blends of polymer semiconductors. Interpenetrating and bicontinuous networks appear to be essential for ready ambipolar charge transport [173].

Bornoz et al. [178] prepared a thin-film photoelectrode via two different deposition techniques: dip coating from a **BBL** solution in MSA [111] and spray deposition of an aqueous **BBL** nanofiber dispersion [114,119]. The photoanodes were used for solar water oxidation in acidic solutions. The results showed a strong dependence of the sacrificial photocurrent density on the morphology of the film, consistent with a limitation in excited-state transport to the direct semiconductor–liquid junction. Both films display a homogeneous coverage of the electrode surface, the dip-coated film shows a relatively smooth surface with feature size similar to the underlying fluorine-doped tin oxide electrode, but the spray-coated film exhibits feature size as small as 20 nm and a higher roughness due to the interconnecting network formed from the nanofiber dispersion. The spray-coated electrode showed the photocurrent density in the *plateau* region (from +1.0−1.3 V v.RHE), 6.5 times higher than dip-coated film. The water oxidation photocurrent density was found to increase with increasing pH, and no evidence of polymer oxidation was found over testing time on the order of hours with bare **BBL** films. This was a first example for application of π-conjugated organic semiconductor to direct solar water oxidation [178].

### 3.6. Photovoltaic Applications

Low production costs, high extinction coefficients, and high electron affinities make the perinones ideal candidates as acceptors in organic photovoltaics (OPV). It should be noted that the concept of heterojunction organic solar cells was developed by Tang in 1985 [152]. Tang showed a bilayer device consisting of **1** as an acceptor and copper(II) phthalocyanine (CuPc) as a donor, arranged in a planar heterojunction array. This type of cell architecture has been shown to reach a power conversion efficiency only nearly 1%, therefore it is used in most cases in bulk heterojunction (BHJ). In BHJ photovoltaic solar cells, appropriate solubilities of both the donor and acceptor compounds in common organic solvents are essential. Due to the low solubility of perinone derivatives, their use in such solar cells has been quite limited. Selected examples are collected in Table 6.

In the case of BHJ, appropriate solubilities of both the donor and acceptor compounds in common organic solvents are essential. In Section 2, it was shown that the flat structures of perinone derivatives are usually difficult to dissolve in typical solvents. The efforts of synthetics to modify their structure in order to improve solubility was also shown. Noteworthy, are the works of Menke et al. who attached three aroyleneimidazole units to the triptycene scaffold to make soluble acceptors, which showed a power conversion efficiency of 3.2% [185]. However, in this case these rigid star-shaped perinone derivatives cannot pack efficiently, which is on the one hand advantageous to increase the solubility but at the same time decreases the density of π-stacking units in the resulting layer after processing, and thus impeding charge transport through the photoactive layer, leading to a reduced fill factor. The highest solar cell efficiency, 7.9%, was obtained for asymmetrical perinone derivative **106 [182]**. The device architecture was an n-i-p type structure, based on a C60/ 2,2′-Bis(2,2-dicyanovinyl)-quinquethiophene (DCV5T)-Me:C60 hybrid planar-bulk heterojunction.

## 4. Summary

Perinon was one of the first synthetic dyes to be used in organic electronics as early as 35 years ago [152]. However, in the following years it was not intensively tested due to its low solubility. The discovery of the soluble Lewis acid coordination complexes of BBL polymer by the Jenekhe Group [186,187] intensified the research on the application of this group of compounds in electronics [74,176,183] and for electrochromism [149]. In recent years, significant progress has been made in the synthesis of soluble perinone derivatives by modifying the naphthalene or perylene core via alkyl [57] or phenyloxy [59] groups substitution. The derivatives of perinone have many advantages that are important in organic electronics applications because they are photochemically very stable and they have high charge mobility. It is also possible to easily modify the width of the band gap. For this reason, they can be the interesting materials in the production of organic transistors, and photovoltaic and light emitting diodes.

## 5. Patents

This section is not mandatory but may be added if there are patents resulting from the work reported in this manuscript.

## Figures and Tables

**Table 1:** Selected examples of condensation reaction components resulting in poly(arylene benzimidazoles).

Dianhydride	Tetramines	Yeld, %	T_dec._, °C	Ref.
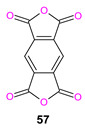	**53**	86–88	450	[85,86]
	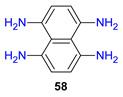	60–90	450	[85]
	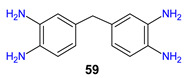	90	440	[87]
	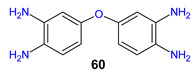	62–95	450	[86,88]
	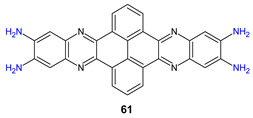	-	450	[89]
**8**	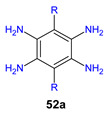	-	300	[90]
	**15a; R = H**	42.5	-	[91]
	**58**	82	400	[85]
	**53**	80	540	[72]
	**59**	95	365	[92]
	**31**	-	-	[56]
	**53**	≈100	470	[93]
	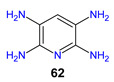	-	-	[94]
	**61**	-	500	[89]
	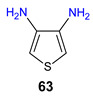	-	-	[95,96]
**12**	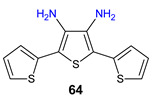	20	-	[64,96]
	**53a**	-	300	[90]
**40**	**53**	85	>400	[97]
	**59**	91	>400	[92]
	**60**	85.5	>400	[92]
	**64**	20	-	[96]
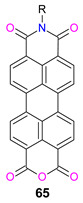	**60**	90	404	[98]
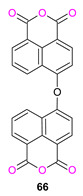	**59**	85	380	[98]
	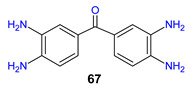	-	>400	[99]
	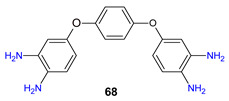	-	>400	[99]
	**53**	~80	>400	[100,101]
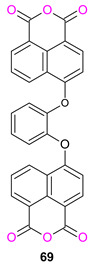	**60**	~80	>400	[100,101]
	**53**	~80	>400	[101]
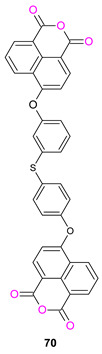	**53**	~80	>400	[101]

**Table 2:** Effect of Beam Current Density (J_b_) on Saturation Conductivity of Implanted **BBL** Films [130].

Implant	Saturation Conductivity (Ω^−1^cm^−1^)	
	J_b_ = 1.0 μA/cm^2^	J_b_ = 2.0 μA/cm^2^
200 keV ^11^B^+^	26	56
200 keV ^40^Ar^+^	74	224
200 keV ^84^Kr^+^	50	136

**Table 3:** Electrochemical properties, and estimated HOMO–LUMO energies of chosen perinone derivatives.

Compound	E_ox_ (V)	E_red_ (V)	HOMO (eV)	LUMO (eV)	Ref.
**1**	−1.03	1.40	−6.20 ^a^ −5.79 ^b^ −6.40 ^c^	−3.78 ^d^ −2.71 ^c^	[129]
**2**	−1.80	1.44	−6.24 ^a^ −5.84 ^b^ −6.47 ^c^	−2.98 ^d^ −2.73 ^c^	[129]
**4**	−1.72	1.32	−6.02 ^a^ −5.86 ^b^ −6.14 ^c^	−3.06 ^d^ −2.73 ^c^	[129]
**6**	−1.61	1.62	−6.06	−3.16	[129]
**13**	−	−0.9	−6.3	−3.9	[143]
**23**	−1.1	−	2.0	1.6 × 10^−5^	[48]
**25**	−1.2	−	−5.8	−3.7	[53]
**26**	−1.1	−	−5.6	−3.6	[53]
**27**	−1.0	−	−6.1	−3.8	[53]
**34**	−1.40	−	−	−	[57]
**74**	−1.1	0.9	−5.21	−3.32	[107]
**80**	−1.13	0.66	−5.32 ^a^ −5.17 ^b^ −5.89 ^c^	−3.81 ^d^ −3.63 ^c^	[129]
**82**	−1.18	1.59	−6.39 ^a^ −5.81 ^b^ −6.51 ^c^	−3.62 ^d^ −2.55 ^c^	[129]
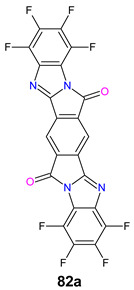	−1.02	−	−6.05 ^a^	−3.82 ^d^	[129]
**83**	−1.24	1.13	−5.31	−3.51	[129]
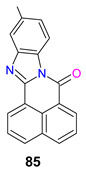	−1.73	1.15	−5.76	−3.06	[129]
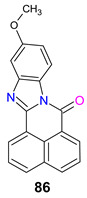	−1.71	0.96	−5.69	−3.07	[129]
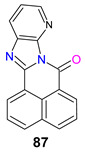	−1.62	1.59	−6.01	−3.16	[129]
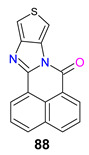	−1.74	1.01	−5.86	−3.04	[129]
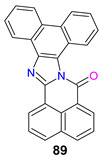	−1.71	0.94	−5.51	−3.08	[129]
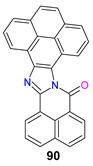	−1.67	0.76	−5.86	−3.11	[129]
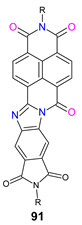	−0.95	−	−6.68 ^b^	−4.00	[144]
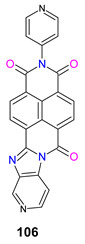	−0.88	−	−6.58 ^b^	−3.90	[144]
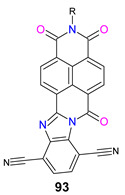	−0.95	−	−5.83 ^b^	−3.79	[144]
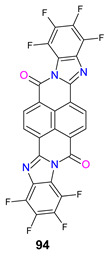	−0.87	−	−6.01 ^a^	−3.92 ^d^	[129]
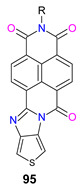	−0.65	1.39	−5.83	−3.79	[95]
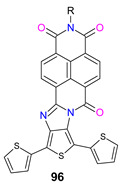	−0.57	1.11	−5.55	−3.87	[95]
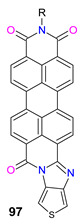	−0.56	−1.32	−5.7	−3.88	[95]
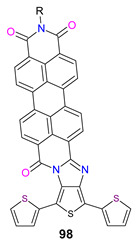	−1.07	0.54	−5.57	−3.85	[64]
	−0.55	1.06	−5.50	−3.89	[95]
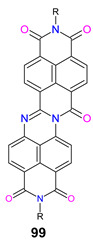	−0.83	−	−5.78	−3.85	[145]

^a^ determined by double pulse voltammetry; ^b^ calculated from the optical energy gap; ^c^ estimated from calculations; and ^d^ determined by cyclic voltammetry.

**Table 4:** Fluorescence quantum yields (Φ_F_), lifetime (τ), band gap (E_g_), and charge mobilities of chosen perinone derivatives.

Molecule	Φ_F_	τ (ms)	E_g_ (eV)	Mobilitiy (cm^2^V^−1^s^−1^)	Ref.
**1**	0.02	4.9	2.01	0.005 ^a^	[129]
**2**	0.04	5.1	2.86	-	[129]
**3**	-	-	1.61	0.0013	[95]
	-	-	1.56	-	[64]
**4**	0.71	11.1	2.8	-	[129]
**23**	-	-	2.0	1.6 × 10^−5^	[48]
	-	-	2.0	0.0013 ^c^	[49]
**26**	-	-	2.1	2.3 × 10^−5^	[54]
**34a** R = nonylphenol	0.9				[163]
**27**	-	-	2.3	2.3 × 10^−5^	[54]
**80**	- ^b^	- ^b^	1.51	-	[129]
				0.05	[164]
**82**	0.02	2.7	2.19	-	[129]
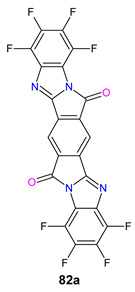	0.11	3.6	2.23	0.027 ^d^	[129]
**91**	0.65	−3.90	2.53	0.0021	[144]
**92**	0.81	−4.00	2.68	0.0016	[144]
**93**	0.45	−3.90	2.68	0.0016	[144]
**94**	0.11	4.0	2.09	0.0013 ^d^	[129]
**95**	-	-	2.04	0.35	[95]
**96**	-	-	1.68	3.5 × 10^−4^	[95]
**97**	-	-	1.88	0.15	[95]
**99**	−5.57	−3.85	1.72	0.05	[145]
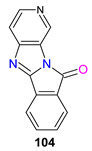	0.58	19.4	3.05	-	[129]
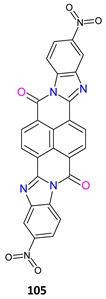	0.51	3.5	2.10	0.0013 ^d^	[129]

^a^ measured in field-effect transistor; ^b^ not estimated due to low solubility, ^c^ annealed at 120 °C for 30 min and then slowly cooled to room temperature, ^d^ thickness: ca. 20 nm.

**Table 5:** n-Channel FET characteristics **BBL**.

Polymer	Thin Film Processing	Buffer Layers	Mobility, μ_e_, (cm^2^V^−1^s^−1^)	I_on_/I_of_	Treshold Voltage, (V)	Ref.
**BBL**	MSA (0.8 wt. %)	-	1.4–5.0 × 10^−4^	30–150	−5	[171]
**BBL**	MSA, FA ^a^ (5:1 *v*/*v*), (0.7 wt.%)	-	2.4 × 10^−5^	8–30	−20	[171]
**BBL**	FeCl_3_,NM ^b^ (0.1 wt.%)	-	9 × 10^−6^	<10	−5	[171]
**BBL**	AlCl_3_,NM ^b^ (n/a)	-	4 × 10^−6^	2–50	n/a	[172]
**BBL**	GaCl_3_,NM ^b^ (n/a)	-	1 × 10^−6^	2–30	n/a	[172]
**BBL**	MSA	-	6.2 × 10^−4^	10^3^	36.5	[170]
**BBL**	MSA	-	8.0 × 10^−4^	10^4^		[173]
**BBL**	Nanobelt network	-	5–7 × 10^−3^	1 × 10^4^	11.9	[114]
**BBL**	MSA, (0.8 wt.%)	Polystyrene	2.2 × 10^−2^	>10^4^	10.93	[174]
**BBL**	MSA	-	3.6 × 10^−4^	>10^3^	~35	[175]
**BBL**	MSA	Polystyrene	1.8 × 10^−2^	>10^3^	~35	[175]
**BBL**	MSA	Benzocyclobutene	1.6 × 10^−2^	>10^3^	~35	[175]
**BBL**	MSA	Ethylene-norborenene copolymer	2.0 × 10^−2^	>10^3^	~35	[175]
**BBL**	MSA	Poly(vinylchloride)	5.2–5.8 × 10^−4^	>10^3^	~35	[175]
**BBL**	MSA	Poly(vinylcarbazole)	5.2–5.8 × 10^−4^	>10^3^	~35	[175]
**BBL**	MSA	Poly(thiazolothiazole)	7.8 × 10^−3^	10^3^	10.1	[176]
**BBL**	MSA	poly(benzobisthiazole-alt-3-octylquarterthiophene)	5.1 × 10^−3^	10^3^	4.3	[176]
**BBL**	MSA ^c^	1,1,1,3,3,3-hexamethyldisila-zane	0.1	2 × 10^3^	10	[74]
**BBB**		1,1,1,3,3,3-hexamethyldisi-lazane	10^−6^	>10^4^	-	[74]
**BBB**	MSA, (0.8 wt.%)	Polystyrene	1.75 × 10^−3^	>10^4^	14.21	[174]
**BBL/BBB** blend	MSA	1,1,1,3,3,3-hexamethyldisi-lazane	10^−5^	>10^4^	-	[74]
**BBL**/PTQ ^d^ blend	MSA	-	1.0 × 10^−3^	200	-	[173]
**BBL**/POT ^e^ blend		-	8.0 × 10^−4^	200	-	[173]

^a^ Formic acid; ^b^ nitromethane; ^c^ annealed at 100–125 °C, 10–30 min; ^d^ PTQ—poly[(thiophene-2,5-diyl)-alt-(2,3-diheptylquinoxaline-5,8-diyl)]; ^e^ POT—poly(10-hexylphenoxazine-3,7-diyl-alt-3-hexyl-2,5-thiophene).

**Table 6:** Physicochemical data of photovoltaic cells based on perinone derivatives.

Perinone Acceptor	Donor	J_SC_ [mA/cm^2^]	V_OC_ [V]	FF	PCE [%]	Ref.
**1**	CuPc	2.3	0.45	0.65	1	[152]
**1**	ZnPc ^a^	2.11	0.5	0.51	0.54	[179]
**13**	PTB7 ^b^	3.6	0.82	0.4	1.2	[143]
**30**	PTB7	4.47	0.90	0.45	1.8	[55]
**30a**	PTB7	5.3	0.96	0.39	2.0	[55]
**37**	CuPc ^c^	2.3	0.45	0.65	0.95	[152]
		4.18	0.4	-	1.1	[180]
			0.54	0.57	2.4 ^d^	[153]
**38a**	ZnPc	4.9	0.48	0.56	1.3	[181]
	CuPc	3.66	0.4	-	0.93	[180]
**74**	PCBM, 1:1	0.60	0.82	0.253	0.125	[107]
	PCBM, 1:2	0.55	1.21	0.267	0.178	
	PCBM, 1:3	0.60	4.00	0.254	0.612	
	PCBM, 1:4	0.55	4.46	0.271	0.666	
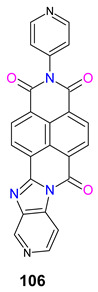	C60/DCV5T ^e^ -MeC60	12.5	0.96	0.657	7.9	[182]
**BBL**	PPV	1.2	1.2	0.41–0.43	0.7–1.2	[167]
		0.182	0.54	0.47	4.6 ^f^	[183]
**BBL**	MEH-PPV	0.144	0.62	0.41	3.7 ^c^	[183]
**BBL**	P3OT	-	0.9	0.023	0.2	[184]

^a^ zincphthalocyanine; ^b^ (poly[[4,8-bis[(2-ethylhexyl)oxy]benzo[1,2-b:4,5-b′]dithiophene-2,6-diyl] [3-fluoro-2-(2-ethylhexyl)-carbonyl]thieno[3,4-b]thio-phenediyl]). ^c^ copper phthalocyanine; ^d^ an additional layer of bathocuproine was applied; ^e^ 2,2′:5′,2″:5″,2‴:5″′,2″″-quinquethio-phene-5,5″″-diyl)bis(methanylylidene)) dimalono-nitrile; and ^f^ annealing temperature 100 °C.

## Data Availability

All data presented in this study is available in M. Lapkowski.
